# Understanding the Effect of Oxygen on M_5_AX_4_ Structure, Stability, and Mechanical Properties

**DOI:** 10.1021/acs.chemmater.5c02254

**Published:** 2025-12-19

**Authors:** Marley Downes, Martin Dahlqvist, Paweł Piotr Michałowski, Johanna Rosen, Yury Gogotsi

**Affiliations:** † Department of Materials Science and Engineering, and A.J. Drexel Nanomaterials Institute, 6527 Drexel University, Philadelphia, Pennsylvania 19104, United States; ‡ Materials Design Division, Department of Physics, Chemistry and Biology (IFM), 4566 Linköping University, Linköping SE-581 83, Sweden; § 196410 Łukasiewicz Research Network—Institute of Microelectronics and Photonics, Warsaw 02-668, Poland; ∥ Wallenberg Initiative Materials Science for Sustainability (WISE), Linköping University, SE-581 83 Linköping, Sweden

## Abstract

M_5_X_4_, the newest and thickest structures in the MXene family, shows promise as mechanically robust nanomaterials. However, the essential role of oxide in their synthesis is poorly understood, which poses a challenge for discovering new M_5_AX_4_ MAX phase precursors. One possibility is that oxygen dissolves into the carbon sublattice, forming stable oxycarbide layers within the MAX phase. Herein, we investigate the layer-by-layer elemental composition of three M_5_AX_4_ compositions: Ti_2.5_Ta_2.5_AlC_4_, Ti_2.675_Nb_2.325_AlC_4_, and Mo_4_VAlC_4_. By modeling the structural stability of each composition, we investigate the possible stabilizing role of oxygen. To guide future application of M_5_X_4_ MXenes, we also calculate the electronic structure and mechanical properties of the parent M_5_AX_4_ MAX phases. This work clarifies the role of oxygen incorporation into MAX phases and its implications for the synthesis and potential applications of their MXene derivatives.

## Graphical abstract

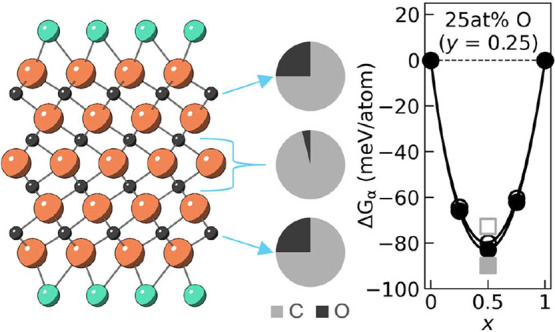

## Introduction

Since the discovery of H-phases (later named MAX phases) in the 1960s, their compositional diversity has grown almost exponentially in the last 10 years.^1^ The initial discovery of Ti_2_SC and Zr_2_SC was soon followed by the discovery of Ti_3_SiC_2_ and Ti_3_GeC_2_, giving rise to the M_*n*+1_AX_*n*_ formula, where M is an early transition metal, A is typically a group 13/14 element, X is carbon and/or nitrogen, and *n* is an integer referring to the number of atomic X-layers.^2–4^ The well-known MXene precursor Ti_3_AlC_2_ was discovered in 1994, further expanding the compositional variety.^5^ The M_4_AX_3_ structure (*n* = 3) was reported shortly after in 1999 with the discovery of Ti_4_AlN_3_.^6^ Beyond these ternary MAX phases, more complex structures have emerged, including solid solutions, intergrown MAX phases, in- and out-of-plane chemical order, double A-layer MAX phases, resulting in ∼350 distinct phases of wide compositional and crystallographic variety.^1^ However, the elusive *n* = 4, or M_5_AX_4_ MAX phases, were not isolated until 2020 when Deysher et al. found that a small amount of V_2_O_3_ was required to synthesize Mo_4_VAlC_4_.^7^ Similarly, Downes et al. showed that TiO_2_ and Nb_2_O_5_ were required for the synthesis of Ti_2.5_Ta_2.5_AlC_4_ and Ti_2.675_Nb_2.325_AlC_4_, respectively.^8^

Alongside the explosion of reported compositions, different synthesis pathways have been developed to produce various MAX phases. Powder metallurgy,^9,10^ thin film deposition,^11,12^ hot-pressing,^13–15^ spark plasma sintering,^16^ and self-propagating high-temperature synthesis (SHS)^17,18^ are among methods used.^1^ A variety of precursors have been utilized, from elemental powders (i.e., Ti + Al + C) to carbides (i.e., Mo_2_C + Ga) and oxides (V_2_O_5_ + Al + C).^9,17,19^ However, of all the MAX phases synthesized to date, only one family requires the addition of an oxide to approach phase-purity: M_5_AX_4_ MAX phases. This raises a crucial question: why is the addition of a small amount of oxide (∼5 at %) necessary to produce this phase? While oxides have been used to produce other MAX phases (as in the previously mentioned SHS synthesis of V_2_AlC^17^), there are alternative nonoxide synthetic pathways available.^20,21^ In the case of M_5_AX_4_, the lack of oxide in the reaction mixture results in the formation of lower *n* MAX phases (*n* < 4) or carbides, depending on the composition.^8,22^ While Mo_4_VAlC_4_ has been synthesized without oxide, the process requires several additional heating steps to produce nonphase-pure (Mo_0.75_V_0.25_)_5_AlC_4_.^23^

M_5_AX_4_ MAX phases are the parent ceramics to M_5_X_4_ MXenes, which stand apart as the thickest and the most rigid yet-synthesized two-dimensional (2D) material. With 11 atomic layers and a thickness of about 1.3–1.4 nm, the M_5_X_4_T_*x*_ structure shows promise in structural applications, composites, sensing, and in nanorobotics that require robust, stiff components.^24^ Additionally, the family displays broad crystallographic diversity, from a unique twinned “herringbone” style structure found in Mo_4_VAlC_4_ to derivatives of the classic *P*6_3_/*mmc* MAX phase structure found in Ti_2.5_Ta_2.5_AlC_4_ and Ti_2.675_Nb_2.325_AlC_4_.^8,23^ The potential incorporation of oxygen into the X-layers further broadens the scope of their compositional complexity.^25,26^

To clarify the role of oxygen, we herein examine the structural stability of three synthesized M_5_AX_4_ MAX phases: Mo_4_VAlC_4_, Ti_2.5_Ta_2.5_AlC_4_, and Ti_2.675_Nb_2.325_AlC_4_. Using Secondary Ion Mass Spectrometry (SIMS), we find that oxygen is preferentially located in the outer X-layers. With this experimental basis, we model M_5_AX_4_ structures, varying both crystal structure as well as oxygen content and distribution to examine any stabilizing effects. Finally, to assess the effect of oxygen on material properties, we calculate the electronic structure and mechanical properties to guide the application of M_5_AX_4_ MAX phases and their related MXenes.

## Results

One possible role of the oxide is to stabilize the thick M_5_AX_4_ structure through oxygen incorporation into the carbon (X) layers. It has been shown that Ti_3_AlC_2_ incorporates oxygen into its carbon sublattice, and this is represented by the formula Ti_3_Al­(C_1–*y*_O_*y*_)_2_, with *y* up to 0.66.^25,26^ Thus, to probe the atomic lattice layer-by-layer, SIMS analysis was conducted on Mo_4_VAlC_4_, Ti_2.5_Ta_2.5_AlC_4_, and Ti_2.675_Nb_2.325_AlC_4_ (Figures 1, S2, and S3). From this data, it is immediately apparent that all examined M_5_AX_4_ MAX phases are oxycarbides. Approximately 30 to 40 at % of the outer carbon sublattice is occupied by oxygen, while the inner X-layers contain a minimal amount of oxygen (<5 at %), making M_5_AX_4_ the first example of ordering on the X-site. It is also apparent that the two metals are found in all five metal layers, albeit with various ratios (Tables S1–S3). Thus, Mo_4_VAlC_4_, Ti_2.5_Ta_2.5_AlC_4_, and Ti_2.675_Nb_2.325_AlC_4_ are most accurately represented by the formulas (Ti_0.5_Ta_0.5_)_5_Al­(C_1–*y*_O_*y*_)_4_, (Ti_0.535_Nb_0.465_)_5_Al­(C_1–*y*_O_*y*_)_4_, and (Mo_0.8_V_0.2_)_5_Al­(C_1–*y*_O_*y*_)_4_, respectively. Measured samples will henceforth be referred to by the (M’M”)_5_Al­(C_1–*y*_O_*y*_)_4_ convention, whereas (M’M”)_5_AlC_4_ will be used for unmeasured samples or theoretical oxygen-free structures.

**Figure 1:**
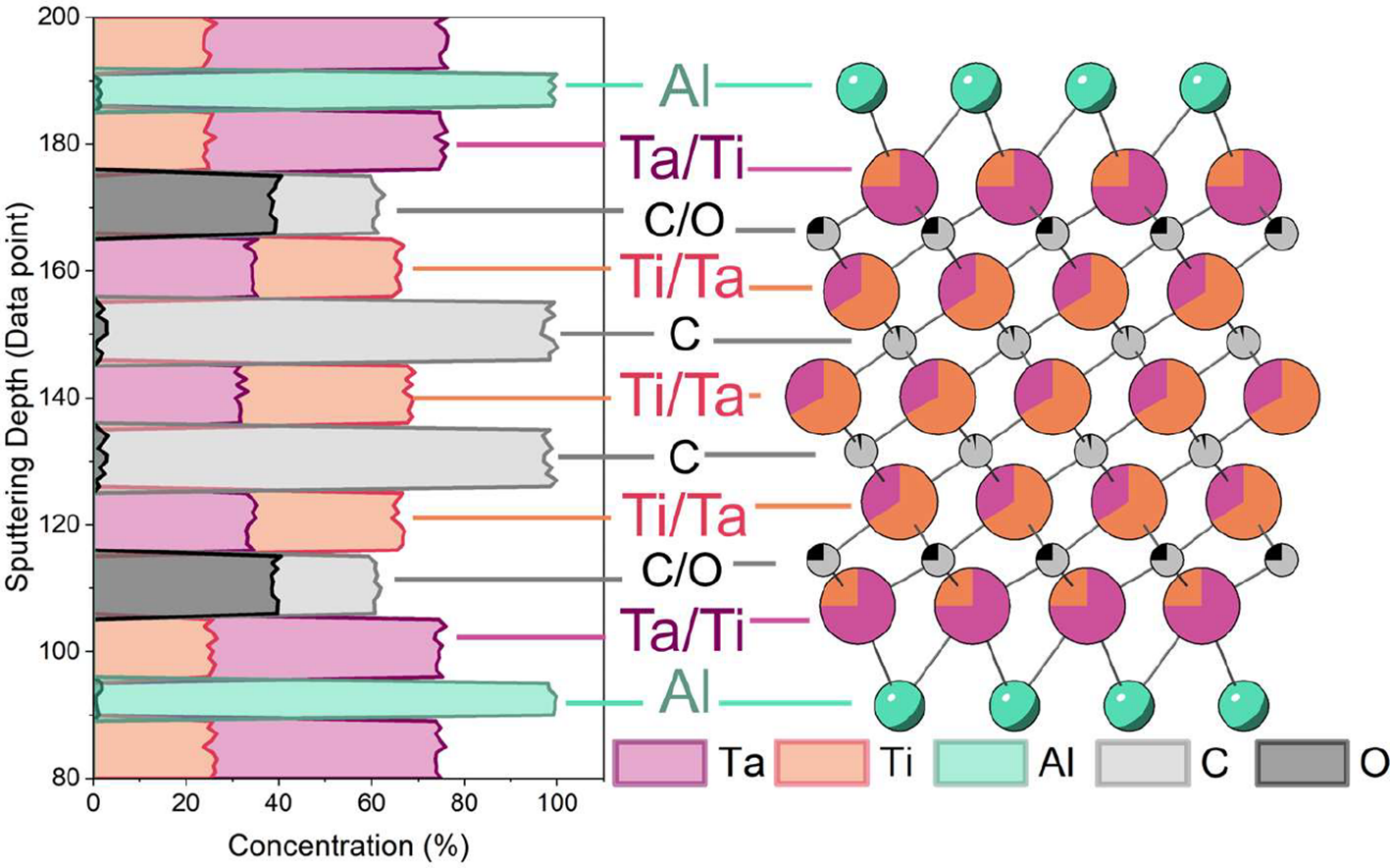
Secondary ion mass spectrometry (SIMS) measurements of (Ti_0.5_Ta_0.5_)_5_Al­(C_1–*y*_O_*y*_)_4_. The M_5_AX_4_ cell illustrates the distribution of elements in the structure.

In contrast to the M_3_AX_2_ MAX phase, where all X-layers are near-surface and susceptible to oxygen incorporation, the M_5_AX_4_ MAX phase has outer near-surface X-layers that can act as an oxygen scavenger, thus protecting the inner subsurface X-layers. This suggests that thicker MAX phase structures may provide a pathway to incorporate transition metals in MAX phases, and thus MXenes, that would otherwise be unstable as oxycarbides by embedding and protecting them within the core of the structures. Interestingly, the recently reported high-entropy M_4_AX_3_ MAX phases do not share this X-site ordering and exhibit a uniform distribution of oxygen.^27^ In the work of Downes et al., discrepancies between the EDS and XPS data suggested partial ordering of the M-elements in the M_5_AX_4_ structure, with elements of larger atomic size, such as Ta, occupying the outer M-layers (in the case of (Ti_0.5_Ta_0.5_)_5_Al­(C_1–*y*_O_*y*_)_4_).^8^ Here, this hypothesis is confirmed, with the trend being most pronounced in (Ti_0.5_Ta_0.5_)_5_Al­(C_1–*y*_O_*y*_)_4_ (Figure 1), where Ta preferentially occupies ∼75% of the outer M-layers and Ti primarily occupies ∼67 at% of the interior M-layers (Table S1). Combined with the presence of oxygen only in the outer X-layers, this suggests that the ordering could be a result of strain minimization to stabilize the structure.^27,28^ To validate this claim, we calculate the stability of all proposed M_5_AX_4_ structures and the resultant influence of oxygen incorporation.

### Structure

The M_5_AX_4_ phase has previously been reported to have different structures, from the traditional MAX phase structure (*P*6_3_/*mmc* space group symmetry),^22^ herein denoted as α-stacking (Figure 2A), to structures where the center M-layer is a twinned plane (*P*6̅*m*2), forming a herringbone-like structure (Figure 2B). The latter has been reported for Mo-rich (Mo_1–*x*_V_*x*_)_5_AlC_4_ with *x* = 0.2 and 0.25.^7,23^ However, the structures reported for (Mo_1–*x*_V_*x*_)_5_AlC_4_ (more precisely, (Mo_0.8_V_0.2_)_5_Al­(C_1–*y*_O_*y*_)_4_) have different positions assigned for Al. Deysher et al. reported Al in a closed-packed face-centered cubic (fcc) position with respect to the M_5_X_4_ subunit. This structure is shown in Figure 2B and denoted α-twinned. Having the fcc A-site is by far the most common A-site position for MAX phases. In contrast, Snyder et al. assigned Al to a hexagonally closed-packed (hcp) position with respect to the M_5_X_4_ subunit (*P*6̅*m*2). This structure is shown Figure 2E and denoted β-twinned. These three structures (α, α-twinned, β-twinned) have been the focus for the M_5_AX_4_ phases studied herein. Three additional structures resembling them have also been considered, which differ in the positions of their Al- and/or X-layer with respect to the M_5_X_4_ subunit. These are illustrated in Figure 2C,D,F and denoted γ, β, and δ, respectively.

**Figure 2:**
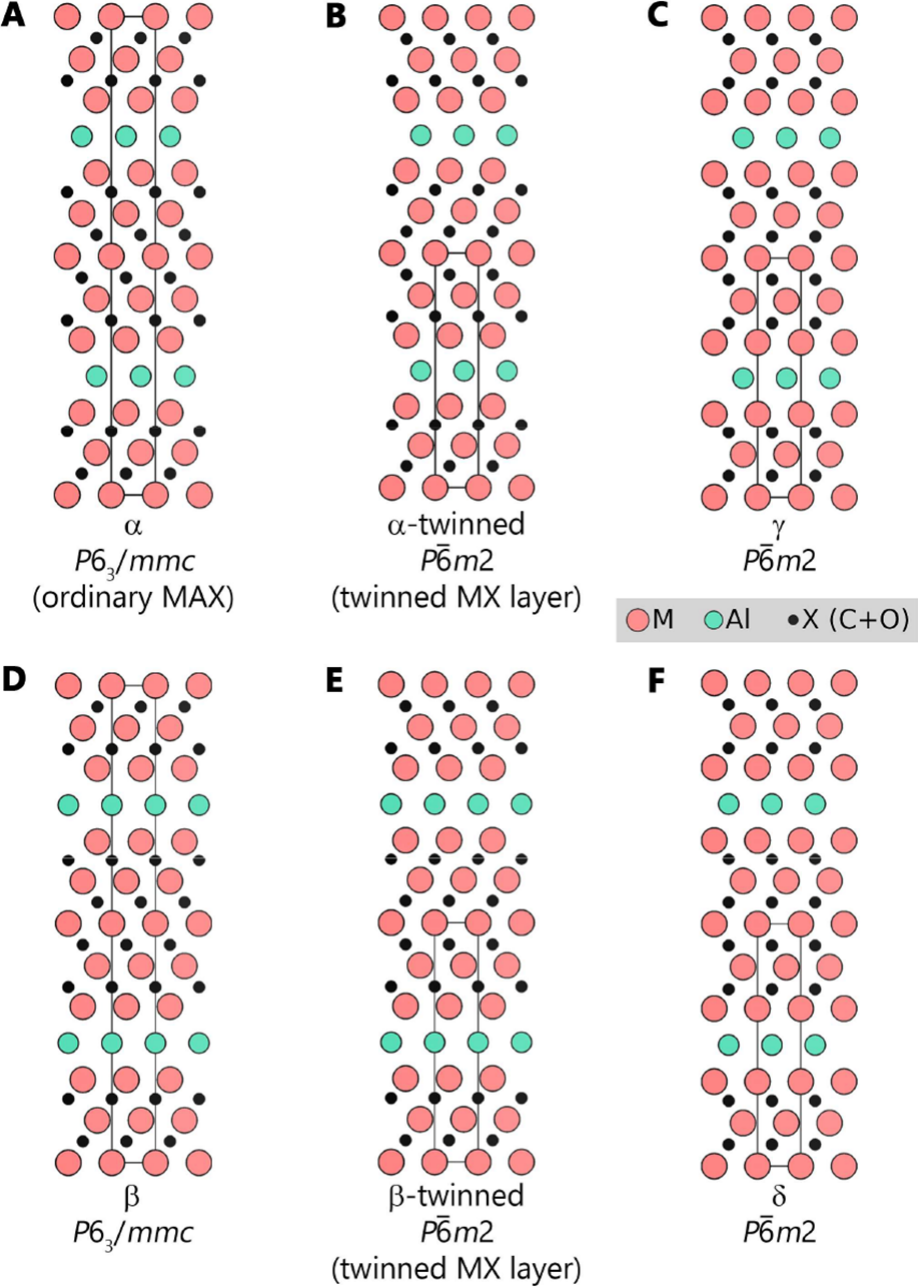
Schematic illustration of structures considered for M_5_AlX_4_. (A) α-stacking corresponding to the traditional MAX phase structure, (B) α-stacking with twinned MX-layers, (C) γ-stacking, (D) β-stacking, (E) β-stacking with twinned MX-layers, and (F) δ-stacking. Structures (A–C) have the A atom in the same in-plane position as the 2nd nearest M-layer, whereas structures (D–F) have the A atom in the same in-plane position as the nearest X-layer. Metal M atoms in red, Al atoms in turquoise, and X in black.

### Stability

Next, we compare the relative stability of the different structures in Figure 2 by evaluating the energy difference, Δ*E*_α_, with respect to the most common MAX phase structure (α, Figure 2A), as defined in eq 1. Figure 3 shows Δ*E*_α_ for 13 ternary M_5_AX_4_ compositions. The α-stacking is lowest in energy for M = Ti, Zr, Hf, and V. For M = Sc, Y, Nb, Ta, Cr, and Mn, the twinned α-stacking (Figure 2B) is found to have the lowest energy, although the α-stacking is close in energy for Nb, Ta, and Cr. The γ-structure (Figure 2C) is lowest in energy for M = Mo and W, whereas the δ-structure (Figure 2F) is most favored for M = Fe. A common feature for each system, except for Fe_5_AlC_4_, is that the lowest energy structure has Al in an fcc position with respect to the M_5_X_4_ subunit, i.e., structures denoted α-, α-twinned, or γ.

**Figure 3:**
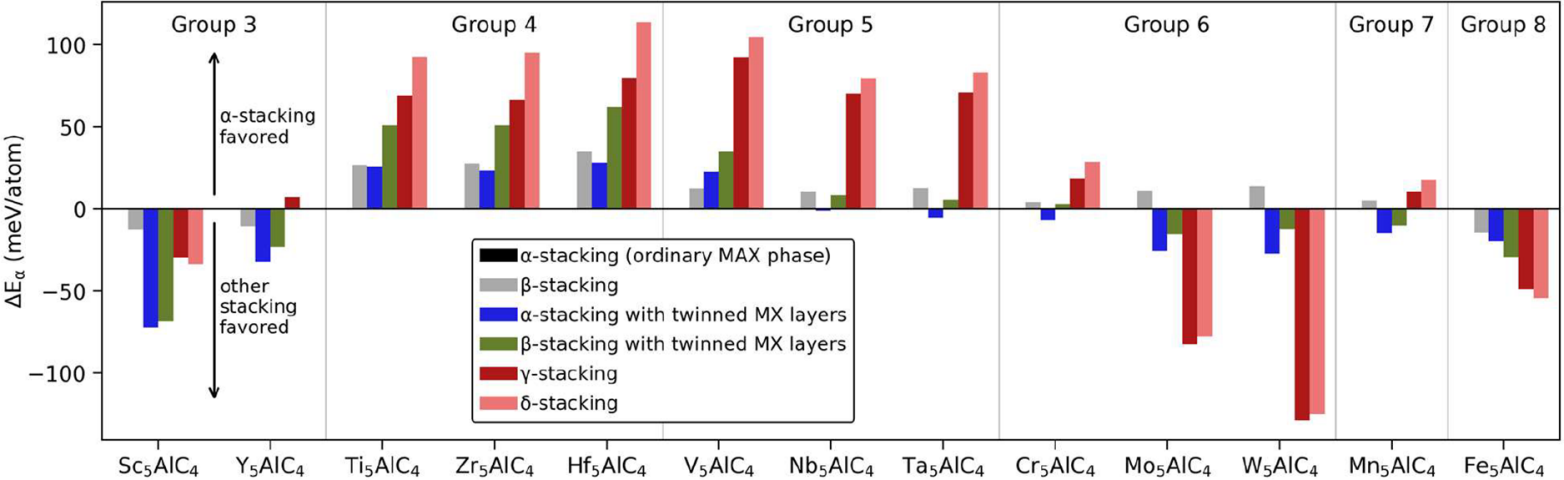
Relative stability for different stackings of M_5_AlC_4_ with respect to the α-stacking.

From the SIMS results, it was observed that for (Ti_0.5_Ta_0.5_)_5_Al­(C_1–*y*_O_*y*_)_4_, (Ti_0.535_Nb_0.465_)_5_Al­(C_1–*y*_O_*y*_)_4_, and (Mo_0.8_V_0.2_)_5_Al­(C_1–*y*_O_*y*_)_4_, oxygen preferentially occupies the X-layers closest to Al. Similar site-dependence was also observed in the metal layers for (Ti_0.5_Ta_0.5_)_5_Al­(C_1–*y*_O_*y*_)_4_, with Ta-rich layers next to the Al-layer and Ti-rich central layers. Previous studies on (Mo_0.75_V_0.25_)_5_AlC_4_ reported a stronger Mo presence on the outer (next to Al) and central M-layers, agreeing with the results in Figure S3.^23^ To investigate how site-dependent occupation, or lack thereof, impacts stability and properties of (M′_1–*x*_M″_*x*_)_5_Al­(C_1–*y*_O_*y*_)_4_, different distributions of M′ and M″ on the metal sublattices and C and O on the X sublattices have been considered. Figures S5 and S6 show the average distribution within each considered layer for 240-atom supercells. Note that both uniform (ideal solid solution) and nonuniform (site-dependent occupation) distributions were modeled. The structures considered for these calculations were α-, α-twinned, γ, and β-twinned, motivated by either (i) being among the lowest energy structures (Figure 3), or (ii) being experimentally reported as M_5_AX_4_ phase structures.^7,8,22,23^

The stability for (M′_1–*x*_M″_*x*_)_5_Al­(C_1–*y*_O_*y*_)_4_ has been evaluated relative to the MAX phase α-structure M_5_AlC_4_ or M_5_Al­(C_1–*y*_O_*y*_)_4_. Since we study a mixture of elements on metal and/or X-sites, the contributions from configurational entropy of mixing (Δ*S*) to the free energy (*G*) will be important at elevated temperatures. This has been accounted for by replacing the *E* terms in eq 1 with *G* as defined in eq 2. Figure 4 shows the relative stability evaluated at 1500 K for *y* = 0 (no oxygen) and *y* = 0.25 (25 at % O on X-sites). Results at additional temperatures (from 0 to 2000 K) and with *y* = 0.167 (16.7 at % O) are shown in Figures S7–S11.

**Figure 4:**
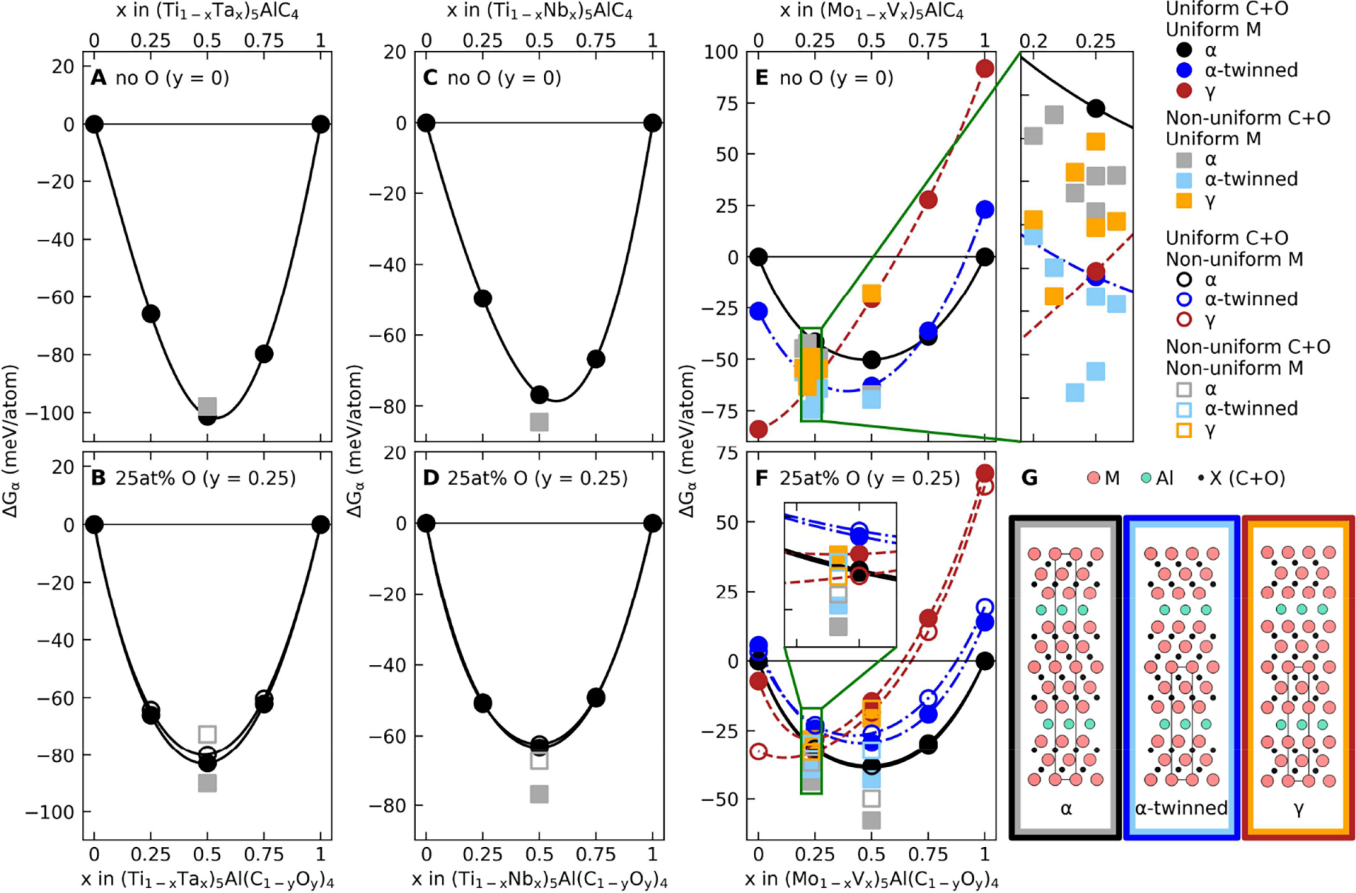
Relative stability in terms of isostructural formation free energy, i.e., with respect to total energy of α-stacking of M_5_AlC_4_ or M_5_Al­(C_1–*y*_O_*y*_)_4_, evaluated at 1500 K for (Ti_1–*x*_Ta_*x*_)_5_Al­(C_1–*y*_O_*y*_)_4_ with (A) no oxygen and (B) 25 at % oxygen; (Ti_1–*x*_Nb_*x*_)_5_Al­(C_1–*y*_O_*y*_)_4_ with (C) no oxygen and (D) 25 at % oxygen; and (Mo_1–*x*_V_*x*_)_5_Al­(C_1–*y*_O_*y*_)_4_ with (E) no oxygen and (F) 25 at % oxygen. Inset of (E, F) shows data around (Mo_0.75_V_0.25_)_5_AlC_4_ for multiple structures with different nonuniform distributions of Mo and V. (G) Schematic illustration of structures whose data are shown in panels (a–f) with metal M atoms in red, Al atoms in turquoise, and X in black.

For (Ti_1–*x*_Ta_*x*_)_5_Al­(C_1–*y*_O_*y*_)_4_ and (Ti_1–*x*_Nb_*x*_)_5_Al­(C_1–*y*_O_*y*_)_4_, we focused on the α-structure. This is justified by calculations showing other structures at higher energies (Figures S12 and S13), and also aligns with previous experimental data.^8^ Both MAX phase systems exhibit a clear energy minimum around *x* = 0.5, with and without oxygen, which agrees with the experimentally observed compositions at *x* = 0.5 for the Ti–Ta and *x* = 0.465 for Ti–Nb. For Ti–Ta with no oxygen (*y* = 0, Figure 4A), both uniform and nonuniform distributions of Ti and Ta result in similar stabilities. When oxygen is introduced (*y* = 0.25, Figure 4B), the most stable configuration has a uniform distribution of oxygen combined with a nonuniform distribution of the metals. For the Ti–Nb system (Figure 4C,D), a nonuniform distribution of metals is slightly favored over uniform by ∼10 meV/atom. The incorporation of oxygen in the X-layers has a small but noticeable contribution to the relative stability for both Ti–Ta and Ti–Nb, indicated by the upward shift of the curves in the bottom panels for 25 at % oxygen (Figure 4B,D) compared to the oxygen-free upper panels (Figure 4A,C). However, we find no significant difference in stability between a uniform and nonuniform distribution of oxygen when the metals are uniformly distributed, as seen by the overlapping lines in Figure 4B,D. When the metals are nonuniformly distributed (Ta- or Nb-rich outer layer), a uniform distribution of oxygen is clearly favored, as indicated by the gray squares being lowest in energy in Figure 4B,D.

For the (Mo_1–*x*_V_*x*_)_5_Al­(C_1–*y*_O_*y*_)_4_ system, four structures have been considered. First to note is that the β-stacking with twinned MX-layers (Figure 2E) is always found at higher energies compared to the α, α-twinned, and γ (Figure S14) and is thus excluded in Figure 4E,F for clarity. Without oxygen (*y* = 0, Figure 4E), the lowest energy structure changes with composition. From γ at *x* < 0.23 (Mo-rich), to α-twinned at 0.23 < *x* < 0.75, and α at *x* > 0.75 (V-rich). Of particular interest is the region around *x* = 0.25 (inset of Figure 4E), where a nonuniform site-dependent distribution of Mo and V is clearly favored over uniform, especially for α and α-twinned structures. Focus will be on α-twinned, since it is lowest in energy. At *x* = 0.233 and 0.25, specific nonuniform distributions of Mo and V, corresponding to Figure S5J,K, are favored, both of which feature an outer M-layer (next to Al) occupied solely by Mo, while the three central layers are composed of both Mo and V.

When oxygen is accounted for in (Mo_1–*x*_V_*x*_)_5_Al­(C_1–*y*_O_*y*_)_4_, only minor differences are found when comparing a uniform and nonuniform distribution of C and O (Figure 4F). However, compared to the oxygen-free configuration (Figure 4E), we observe a shift in the lowest energy structure from γ at *x* < 0.25 to α for *x* > 0.25 when Mo and V are uniformly distributed. For a nonuniform metal distribution around *x* = 0.25 and at *x* = 0.5, the α structure is lowest in energy at −37 and −50 meV/atom, corresponding to the distributions shown in Figure S5J,E, respectively. However, the α-twinned is close in energy at −30 and −42 meV/atom at *x* = 0.233 and 0.5, respectively.

Based on Figures 4A–D and S4–S11, we can conclude that for oxygen-free Ti-based systems, mixed with Ta or Nb, uniform and nonuniform metal distributions are close to degenerate in energy. When oxygen is present, nonuniform metal distribution, indicating site-dependent composition, combined with a uniform distribution of C and O is favored (black triangles in Figure 4B,D). However, it should be noted that the difference is small and within 15 meV/atom. For the Mo–V system, nonuniform metal distribution is preferred, at least for *x* = 0.25 and 0.5, but with a structural dependence in the form of preference for α-twinned at *y* = 0 (no oxygen) and α at *y* = 0.25 (25 at % O). Again, a uniform distribution of C and O is always favored. It should be noted that the results shown herein are valid for ideal occupation on all sublattices, and no deviation from full occupancy has been considered, e.g., any vacancies on M-, A, and X-sites. Thus, it can be concluded that the effect of oxygen, at least in stabilizing the overall structure, is negligible compared to the oxygen-free structures. This suggests that the role of oxides in synthesizing M_5_AX_4_ MAX phases is likely kinetic and reaction pathway dependent rather than thermodynamic, where the oxide may facilitate a high-temperature carbothermal reduction.^29,30^ Future studies should utilize in situ high-temperature characterization techniques, such as XRD and TGA/DSC, to examine the reaction pathway both with and without oxide addition to confirm this hypothesis.^23^

### Properties

The electronic structure of (Ti_1–*x*_Ta_*x*_)_5_Al­(C_1–*y*_O_*y*_)_4_, (Ti_1–*x*_Nb_*x*_)_5_Al­(C_1–*y*_O_*y*_)_4_, and (Mo_1–*x*_V_*x*_)_5_Al­(C_1–*y*_O_*y*_)_4_ was investigated in terms of density of state (DOS) calculations to study the impact of oxygen incorporation. Figure 5 shows the total and partial DOS for M- and X-site distributions that best match the SIMS results. For Ti–Ta and Ti–Nb systems, the α-stacking is used, and for Mo–V, the α-twinned structure. All three systems were considered with a nonuniform C and O distribution. Supplementary DOS for other distributions of metals and/or C + O are shown in Figures S15–S19. For both Ti–Ta and Ti–Nb, the Fermi level (*E*_f_) is located near a local minimum, also known as a pseudo gap, which separates bonding states below the minima from non- and/or antibonding states above it. For Mo–V, the additional number of electrons pushes *E*_f_ to higher energies, thus shifting it away from the minima. All systems are found with a finite number of states at *E*_f_, primarily composed of metal d-states that indicate metallic conductivity. It should be noted that mixing metals leads to a more smeared-out DOS and an increased number of states at *E*_f_ as compared to the single-metal M_5_AX_4_ shown in Figure S15. Similarly, when oxygen is incorporated in the X-layers, the DOS also becomes more smeared-out with an increased number of states at *E*_f_ compared to their oxygen-free counterparts (Ti_1–*x*_Ta_*x*_)_5_AlC_4_, (Ti_1–*x*_Nb_*x*_)_5_AlC_4_, and (Mo_1–*x*_V_*x*_)_5_AlC_4_ in Figures S13 and S15. A comparison of DOS for different metals (Figures S16 and S18) and oxygen (Figures S17 and S19) distributions shows that the specific site-occupancy on M- and X sublattices has little impact on the overall DOS for all the systems. The key takeaway is that both metal alloying and oxygen incorporation lead to less distinct peaks in the DOS and an increased number of states at *E*_f_.

**Figure 5:**
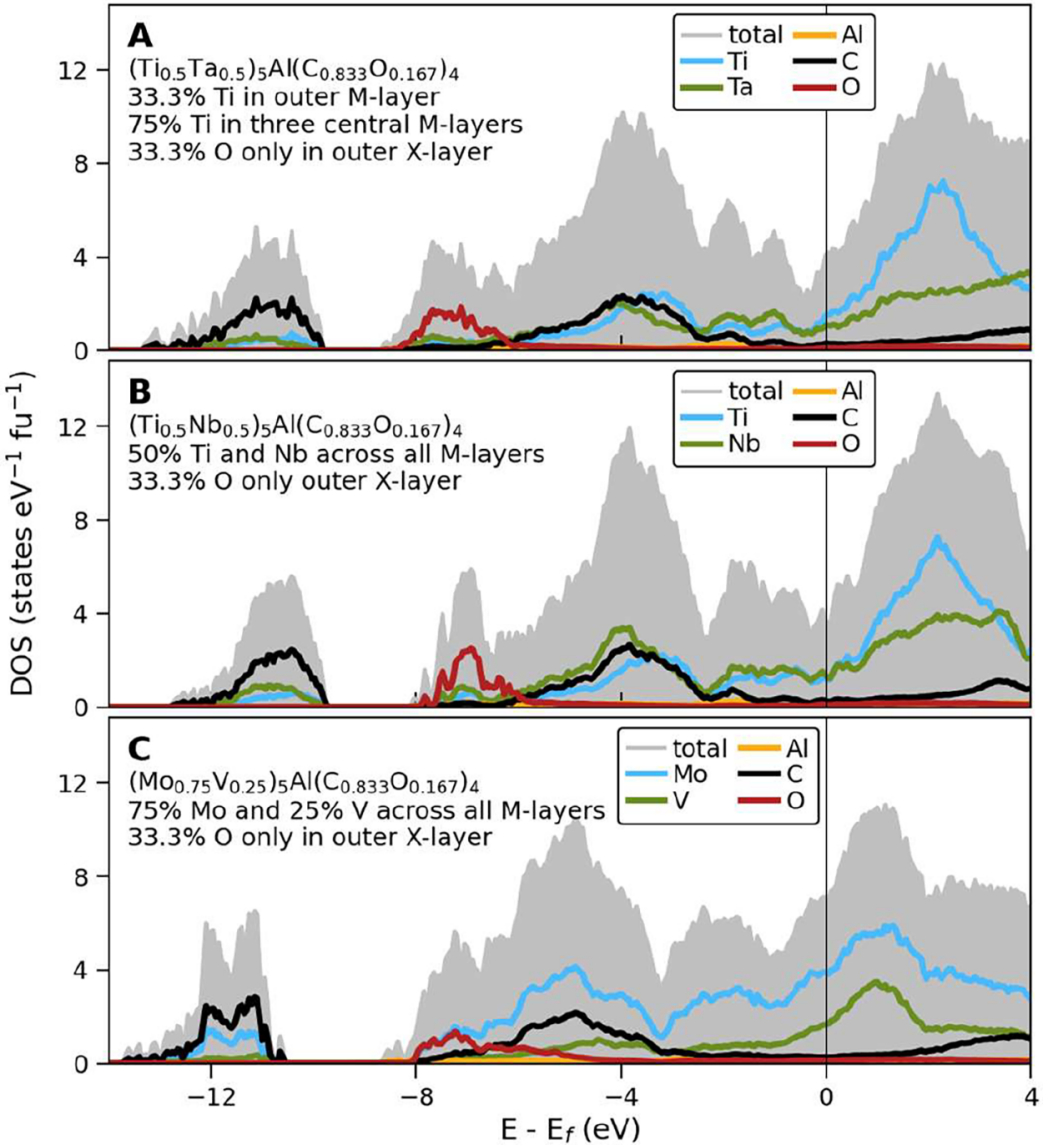
Calculated density of states (DOS) for (A) nonuniform distribution of Ti and Ta in (Ti_0.5_Ta_0.5_)_5_Al­(C_0.833_O_0.167_)_4_ with α-stacking, (B) uniform distribution of Ti and Nb in (Ti_0.5_Nb_0.5_)_5_Al­(C_0.833_O_0.167_)_4_ with α-stacking, and (C) uniform distribution of Mo and V in (Mo_0.75_V_0.25_)_5_Al­(C_0.833_O_0.167_)_4_ with twinned α-stacking. All structures have a nonuniform distribution of oxygen in the X-layers.

Before the discovery of MXenes in 2011, MAX phases had gained significant attention due to their unique combination of physical properties.^31^ Notably, MAX phases retain the elastic stiffness of their corresponding binary carbides but are also unusually soft, damage-tolerant, and possess mobile basal plane dislocations due to their layered nature.^32^ Combined with the potential of their MXene counterparts being applied in nanorobotics, the thick M_5_AX_4_ structure should stand out as the most mechanically robust family of MAX phases.^24^ Thus, we have calculated mechanical properties for selected M_5_AX_4_ compositions based on the SIMS results, comparing oxygen-free (*y* = 0) with a nonuniform distribution of 16.7 at % oxygen (*y* = 0.167). For (Ti_0.5_Ta_0.5_)_5_Al­(C_1–*y*_O_*y*_)_4_, we choose a nonuniform Ti/Ta distribution with a Ta-rich outer layer (Figure S5D), while for (Ti_0.5_Nb_0.5_)_5_Al­(C_1–*y*_O_*y*_)_4_, a uniform Ti/Nb distribution was used (Figure S5B). For (Mo_1–*x*_V_*x*_)_5_Al­(C_1–*y*_O_*y*_)_4_, both uniform (Figure S5A) and nonuniform (Figure S5J) Mo/V distributions were considered. For comparison, M_5_Al­(C_1–*y*_O_*y*_)_4_ with M = Ta, Ti, and Nb have also been included.

Starting with oxygen-free systems, we observe that the elastic (*E*) and shear (*G*) moduli for Ti–Ta and Ti–Nb solid solutions are higher and/or equivalent to the values calculated for corresponding M_5_AX_4_ ternaries. This can be interpreted as a strengthening effect upon alloying. The bulk modulus (*B*) values are close to the average values from the corresponding ternaries. When oxygen is incorporated, the general trend is a decrease of *B*, *E*, and *G*. From this, we can conclude that oxygen tends to soften the M_5_AX_4_ phases studied herein. If this is valid for MAX phases in general remains to be investigated.

Compared with other MAX phases, M_5_AX_4_ retains the trend that stiffness increases with increasing *n*. For instance, in the Ti_*n*+1_AlX_*n*_ system, the experimentally measured Young’s modulus (*E*) increases steadily with increasing *n* (Table 1), a trend that continues for the Ti–Ta and Ti–Nb M_5_AX_4_ systems (Figure 6B). Considering recent insights into the layer-by-layer etching mechanism, the increasingly harsh synthetic methods required to etch higher-*n* MAX phases can be connected to the increased rigidity of the lattice.^33^ As the bulk modulus (*B*) is a measure of compressibility, it is not surprising that increasing *n* would also increase *B*—the thicker the structure becomes, the more rigid the lattice. This aligns with the observation that M_5_X_4_ films are more rigid and brittle.^8^ Similarly, the shear modulus increases slightly with increasing *n,* with the exception of the Mo–V M_5_AX_4_ system. As (Mo_1–*x*_V_*x*_)_5_AlC_4_ (more precisely, (Mo_0.8_V_0.2_)_5_Al­(C_1–*y*_O_*y*_)_4_) exhibits a twinned crystal structure, it may offer less resistance to shear stress than the conventional *P*6_3_/*mmc* structure.^23^ It is important to note that this comparison is between theoretical modeling and experimental measurement; however, past studies have found good agreement between experimentally and theoretically determined moduli.^32^ While the mechanical properties of the MAX phases outperform those of the lower-*n* systems, those of the respective MXenes remain to be explored due to the increased complexity of the system. The influence of defects from the harsh synthetic conditions, small flake size, and random T_*x*_ terminations further confounds experimental measurement or theoretical modeling.^34,35^ To realize the potential of M_5_X_4_ in nanomechanical applications, future work should focus on developing less harsh, gentler synthesis conditions to reduce defects, increase flake size, and improve overall stability.^8,33,36^

**Figure 6:**
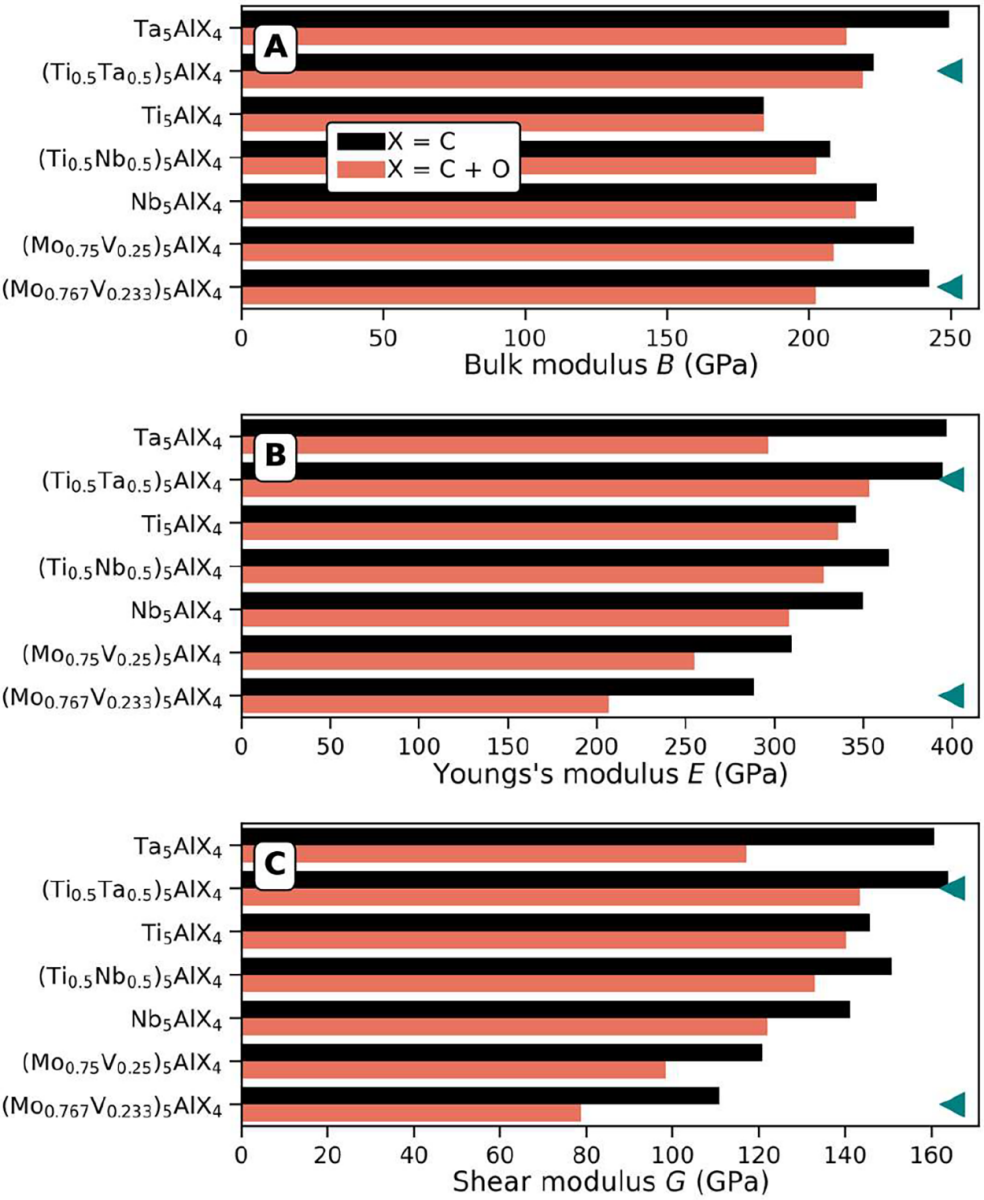
Mechanical calculations of (A) bulk (*B*), (B) Young’s (*E*), and (C) Shear modulus (*G*) for M_5_Al­(C_1–*y*_O_*y*_)_4_ and (M′_1–*x*_M″_*x*_)_5_Al­(C_1–*y*_O_*y*_)_4,_ both with (red, *y* = 0.25) and without (black, *y* = 0) oxygen. Triangles show phases with a nonuniform distribution of metals M′ and M″.

**Table 1:** Selected Experimental Moduli for *n* = 1–3^a^

	M_*n*+1_AX_*n*_	*B* (GPa)	*E* (GPa)	*G* (GPa)	ref
*n* = 1	Ti_2_AlC	186 ± 2	277	118	^37–39^
	Ti_2_AlC_0.5_N_0.5_		290	123	^40^
	Ta_2_AlC	251	292	121	^13^ and^37^
	Nb_2_AlC	208	286	117	^37^ and^41^
	V_2_AlC	201 ± 3	235	116	^37^ and^41^
*n* = 2	Ti_3_AlC_2_	226 ± 3	297.5 ± 2	124 ± 2	^38,42^, and^43^
	Ti_3_AlCN	219 ± 4	330	137	^40^ and^43^
	Ta_3_AlC_2_	238*			^44^
*n* = 3	Ti_4_AlN_3_	216 ± 2	310 ± 2	127 ± 2	^42^ and^45^
	V_4_AlC_3_	218*	353*	170*	^46^
	Nb_4_AlC_3_	214*	306	127	^46^ and^47^
	Ta_4_AlC_3_	261 ± 2	324	132	^48^ and^49^

a Values marked with * are theoretically calculated moduli. Nitride MAX phases are included to extend the scope of *n,* but it is important to note that higher defect concentrations in nitrides tend to lower experimentally measured moduli compared to theoretical prediction.^32^ Experimental bulk moduli values are taken from the anvil cell measurement.

## Conclusions

In this work, we have investigated the role of oxygen in the M_5_AX_4_ system and found that all three investigated MAX phases are ordered oxycarbides, with oxygen preferentially located in the outer X-layers closest to the Al-layer. By evaluating the structural stability of the proposed M_5_AX_4_ structures, we find that (Ti_0.5_Ta_0.5_)_5_Al­(C_1–*y*_O_*y*_)_4_ and (Ti_0.535_Nb_0.465_)_5_Al­(C_1–*y*_O_*y*_)_4_ prefer the traditional *P*6_3_/*mmc* structure, while (Mo_0.8_V_0.2_)_5_Al­(C_1–*y*_O_*y*_)_4_ is most stable in the twinned *P*6̅*m*2 structure, regardless of oxygen content. The negligible change in relative Gibbs free energy upon oxygen incorporation suggests that the role of the oxide precursors during MAX synthesis is to change the kinetics and reaction pathway rather than to thermodynamically stabilize the M_5_AX_4_ structure. Electronic structure calculations show that solid solution alloying on both the M- and X-sites leads to a broadening of the density of states. In terms of mechanical properties, metal-site alloying leads to increased mechanical robustness, while oxygen incorporation into the X-layers leads to a softening of the structure. This work deepens the understanding of the role of oxygen in M_5_AX_4_ MAX phases and corresponding M_5_X_4_T_*x*_ MXenes and suggests that oxygen-free M_5_AX_4_ may be attainable under alternative synthesis routes. The predicted properties herein may guide the future applications of these classes of layered and 2D materials.

## Methods

### Synthesis of M_5_AX_4_ MAX Phases

The synthesis of M_5_AX_4_ MAX phases was done according to previous reports.^7,8^ Briefly, titanium (∼325 mesh, 99.5% metals basis), titanium­(IV) oxide, niobium (∼325 mesh, 99.8% metals basis), niobium (V) oxide (99.5% metals basis), tantalum (∼325 mesh, 99.9% metals basis, 99.6%), molybdenum (∼250 mesh, 99.9% metals basis), vanadium (∼325 mesh, 99.5%), vanadium­(V) oxide (99.2%), aluminum (∼325 mesh, 99.5% (metals basis)), and graphite (∼325 mesh, 99%) powders were combined in the appropriate atomic ratios. The powders were then ball milled at 70 rpm for 18 h at a ratio of 1:2 powder to yttria-stabilized zirconia (YSZ) balls by mass. The powders were then heated in alumina crucibles at 3 °C min^–1^ to 1650 °C under 350 cm^3^ min^–1^ flowing argon in a tube furnace (Carbolite Gero) and held for 4–24 h. The resultant MAX phases were ground to powder using a mortar and pestle, then washed in 9 M HCl before being dried and sieved to <75 μm.

### SIMS

All SIMS experiments were performed on the CAMECA IMS SC Ultra instrument with cesium as primary ions. To reach the atomic depth resolution, a series of modifications of the measurement procedure were applied, including high incident angle bombardment (75°), ultralow impact energy (100 eV), in situ ion polishing, optimization of extraction parameters, super cycle, and advanced beam positioning. The details of each concept were presented in previous work.^25^ Deconvolution and calibration protocols were applied to quantify the results and determine the exact composition of each atomic layer with ±1% precision.^26^

### DFT Calculations

All first-principles calculations were performed by means of density functional theory (DFT) and the projector augmented wave method,^50,51^ as implemented within the Vienna ab initio simulation package (VASP) version 5.4.1.^52–54^ We used the nonspin polarized generalized gradient approximation (GGA) as parametrized by Perdew–Burke–Ernzerhof (PBE) for treating the electron exchange and correlation effects.^55^ The plane wave energy cutoff was set to 520 eV and sampling of the Brillouin zone was performed using the Monkhorst–Pack scheme with a *k*-point density of 0.1 Å^–1^. For each structure. the volume, shape, and internal atomic positions were relaxed until an energy and force convergence of 10^–6^ eV/atom and 10^–2^ eV/Å, respectively, were reached.^56^

Relative stabilities for various stackings considered for the M_5_AX_4_ phase have been quantified in terms of energy difference Δ*E*_α_ by comparing their energy to the energy of the α-structure, according to

$$\Delta E_{\alpha }=E[({M^{’}}_{1-x}{M^{’’}}_{x}{)}_{5}\mathrm{Al}(C_{1-y}O_{y}{)}_{4}]-E_{\alpha }[({M^{’}}_{1-x}{M^{’’}}_{x}{)}_{5}\mathrm{Al}(C_{1-y}O_{y}{)}_{4}]$$ 1

and can be generally used for ternary, quaternary, and quinary compositions depending on the values of *x* and *y.*

To model compositional disorder of M′ and M″ on the metal (M) sublattices and of carbon and oxygen on the X sublattices in (M′_1–*x*_M″_*x*_)_5_Al­(C_1–*y*_O_*y*_)_4_, the special quasi-random structure (SQS) method^57^ was used with supercell sizes consisting of 240 atoms. Figure S4 shows a schematic illustration of the supercells considered for four different structures. Both uniform and nonuniform distributions were considered for different values of *x* (M sublattices) and *y* (X sublattices). This is illustrated in Figures S5 and S6.

For structures with compositional disorder, the contribution from configurational or compositional entropy due to disorder of M′ and M″ on the metal (M) sublattices and/or of C and O on the X sublattices will decrease the Gibbs free energy *G* as approximated by

G[T]=E−TΔS 2

where the entropic contribution Δ*S*, assuming an ideal solution of M′ and M″ on all or selected metal (M) sublattices and of C and O on all or selected X sublattices, is given by

$$\Delta S=-k_{B}\left(\sum_{i=1}^{n}\{w_{i}[x_{i}\ln(x_{i})+(1-x_{i})\ln(1-x_{i})]\}+\sum_{j=1}^{n}\{w_{j}[y_{j}\ln(y_{j})+(1-y_{j})\ln(1-y_{j})]\}\right)$$ 3

where *k*_B_ is the Boltzmann constant, *w*_*i*,*j*_ is the site fraction, i.e., atoms on sublattice *i* and/or *j* with respect to all atoms in the supercell, *x*_*i*_ is the concentration of M″ on the M-sublattice *i*, and *y*_*i*_ is the concentration of O on X-sublattice *j.*

The elastic properties were derived through the use of VASPKIT^58^ and the stress–strain approach. Bulk (*B*), shear (*G*), and Young’s elastic (*E*) modulus are based on the Voight–Reuss–Hill approximation. The presented values herein thus represent the Hill averages.

Illustrations of crystal structures have been made using VESTA^59^ or CrystalMaker.

## Supplementary Material



## Notes

SIMS measurements for (Ti_0.535_Nb_0.465_)_5_Al­(C_1–*y*_O_*y*_)_4_ and (Mo_0.8_V_0.2_)_5_Al­(C_1–*y*_O_*y*_)_4_, and tabulated elemental concentrations for all three M_5_AX_4_ MAX phases; supercell illustrations for various crystal structures; schematics of metal and X-site distributions; and stability calculations and simulated DOS for various crystal structures and elemental compositions and distributions (PDF)
