# Supplementary material for: Understanding the Effect of Oxygen on M5AX4 Structure, Stability, and Mechanical Properties
Source: Chem Mater. 2025 Dec 19;38(1):231–9. doi: 10.1021/acs.chemmater.5c02254 (PMC12805517; doi:10.1021/acs.chemmater.5c02254)
Supplement: Supplementary file 1 [file cm5c02254_si_001.pdf]

Supporting Information

Understanding the effect of oxygen on M_5AX_4 structure, stability, and mechanical properties

Marley Downes[1], Martin Dahlqvist[2], Paweł Piotr Michałowski[3], Johanna Rosen[2, 4], Yury Gogotsi[1, 4*]

[1]Department of Materials Science and Engineering, and A.J. Drexel Nanomaterials Institute, Drexel University, Philadelphia, Pennsylvania 19104, United States

[2]Materials Design Division, Department of Physics, Chemistry and Biology (IFM), Linköping University, SE-581 83 Linköping, Sweden

[3]Łukasiewicz Research Network—Institute of Microelectronics and Photonics, Warsaw, Poland

[4] Wallenberg Initiative Materials Science for Sustainability (WISE), Linköping University, SE-581 83 Linköping, Sweden.

*Corresponding author: gogotsi@drexel.edu

SIMS Analysis

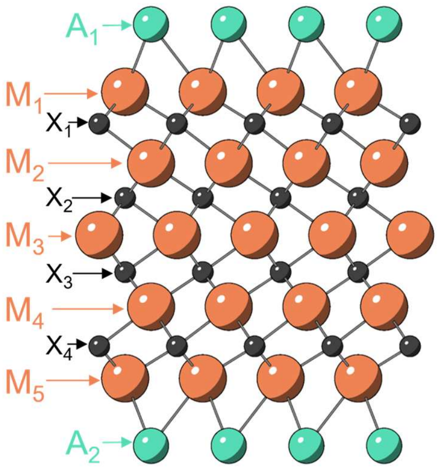

Figure S1. Labeled M_5AX_4 structure showing the names given to each row of atoms as part of the SIMS analysis.

Table S1. Tabulated SIMS-determined compositions (**Figure 1**) by at.% concentration of elements for $(Ti_{0.5}Ta_{0.5})_5Al(C_{1-y}O_y)_4$. Compositions organized by layer with respect to the labeling convention in **Figure S1**.

Layer	Ti	Ta	C	O
$\mathbf{M_{1,5}}$	24.6±0.7	75.4±1.3	–	–
$\mathbf{M_{2,4}}$	66.3±0.7	33.9±1.3	–	–
$\mathbf{M_3}$	67.1±0.7	32.9±1.2	–	–
$\mathbf{X_{1,4}}$	–	–	74.5±0.7	25.6±1.3
$\mathbf{X_{2,3}}$	–	–	96.6±0.7	3.4±1.4

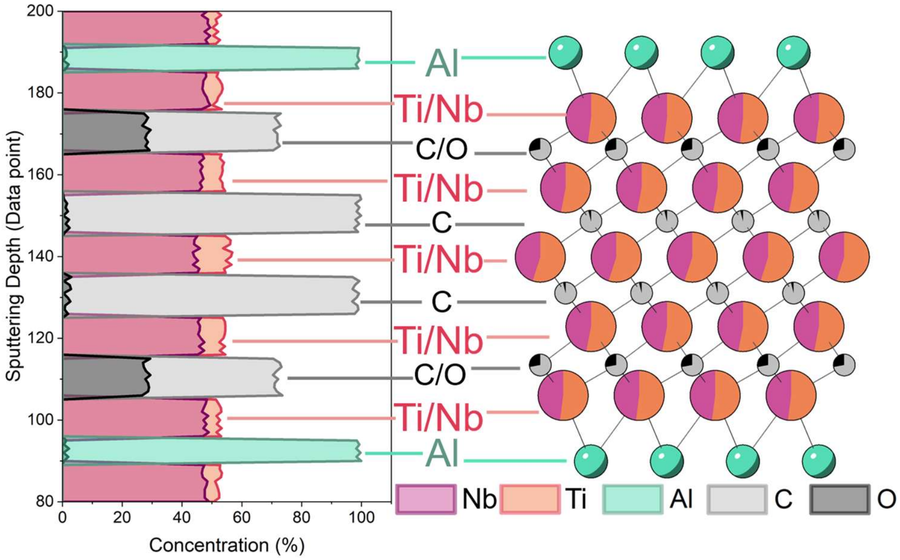

Figure S2. Secondary ion mass spectrometry (SIMS) measurements of $(Ti_{0.5}Nb_{0.5})_5Al(C_{1-y}O_y)_4$. The M_5AX_4 cell illustrates the distribution of elements in the structure.

Table S2. Tabulated SIMS-determined compositions (**Figure S2**) by % concentration of element for $(Ti_{0.5}Nb_{0.5})_5Al(C_{1-y}O_y)_4$. Compositions are organized by layer with respect to the labeling convention in **Figure S1**.

Layer	**Ti**	**Nb**	**C**	**O**
**$M_{1,5}$**	51.5±0.7	48.4±1.3	–	–
**$M_{2,4}$**	53.4±0.7	46.5±1.4	–	–
**M_3**	55.4±0.7	44.7±1.3	–	–
**$X_{1,4}$**	–	–	72.0±0.7	28.0±1.4
**$X_{2,3}$**	–	–	96.4±0.7	3.6±1.4

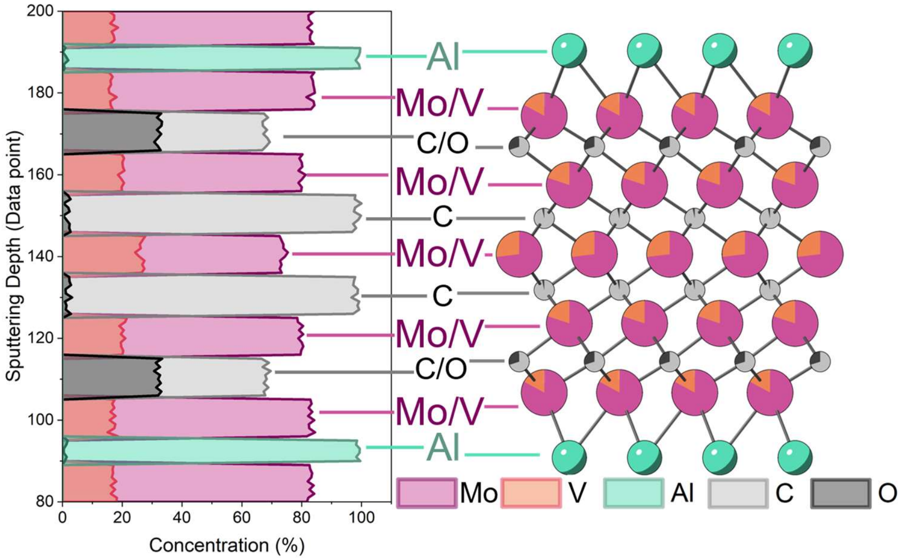

Figure S3. Secondary ion mass spectrometry (SIMS) measurements of $(Mo_{1-x}V_x)_5Al(C_{1-y}O_y)_4$. The M_5AX_4 cell illustrates the distribution of elements in the structure.

Table S3. Tabulated SIMS-determined compositions (**Figure S3**) by % concentration of element for $(Mo_{1-x}V_x)_5Al(C_{1-y}O_y)_4$. Compositions are organized by layer with respect to the labeling convention in **Figure S1**.

Layer	**Mo**	**V**	**C**	**O**
**$M_{1,5}$**	82.9±0.7	17.0±1.3	–	–
**$M_{2,4}$**	80.1±0.7	19.9±1.4	–	–
**M_3**	73.1±0.6	26.9±1.2	–	–
**$X_{1,4}$**	–	–	70.0±0.7	29.9±1.3
**$X_{2,3}$**	–	–	96.5±0.7	3.4±1.4

Supercells and distribution of atoms in M- and X-sublattices

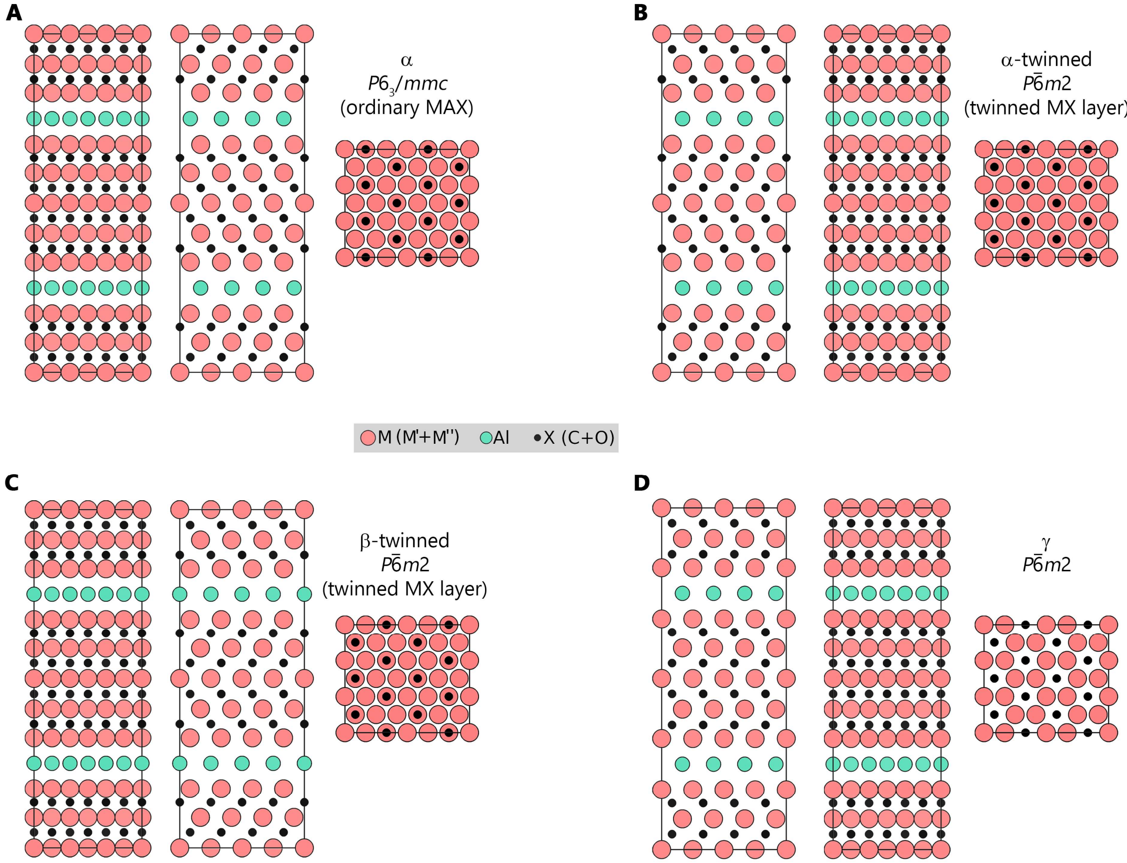

Figure S4. Schematic illustration of 240-atom supercells considered for (**A**) α-stacking corresponding to the ordinary MAX phase structure, (**B**) α-stacking with twinned MX-layers, (**C**) β-stacking with twinned MX-layers, and (**D**) γ-stacking, viewed along two in-plane directions and one top view. Metal M atoms in red, Al atoms in turquoise, and X in black.

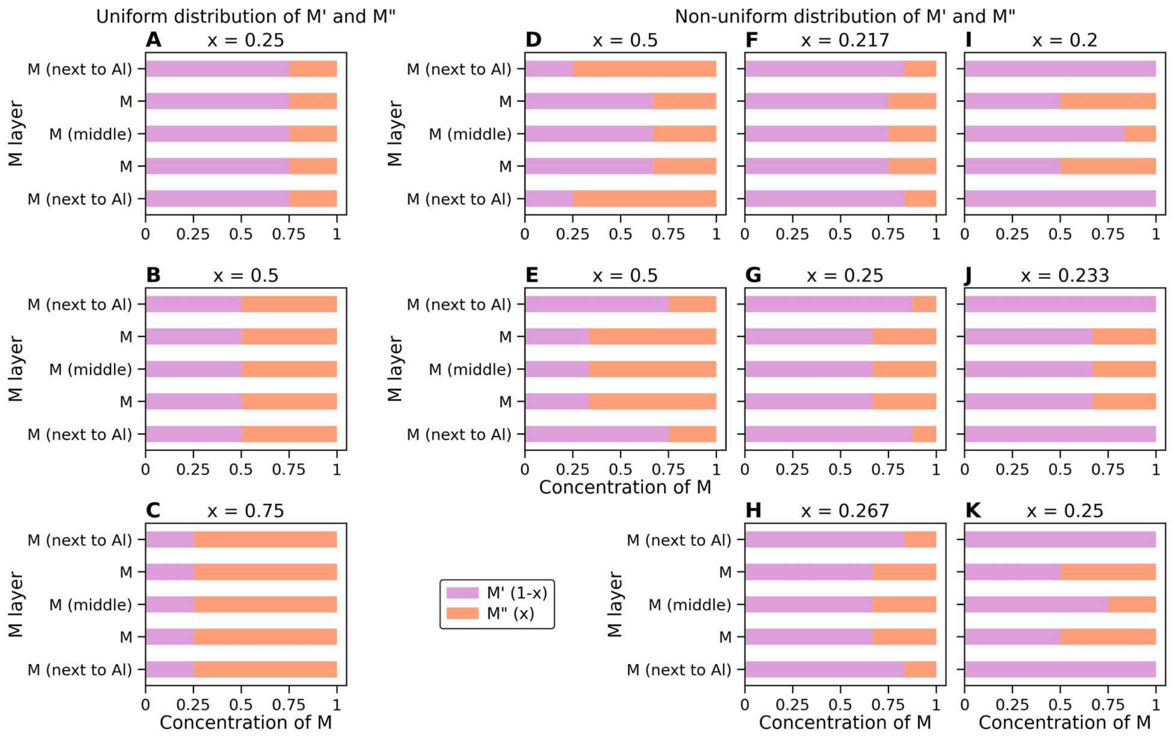

Figure S5. Schematic illustration of the considered distributions of metals. (**A** to **C**) Uniform and (**D** to **K**) non-uniform, i.e., site dependent, distribution of metals M' and M'' for different concentration x of M'' in $(M'_{1-x}M''_{x})_5AlC_4$ and $(M'_{1-x}M''_{x})_5Al(C_{1-y}O_{y})_4$.

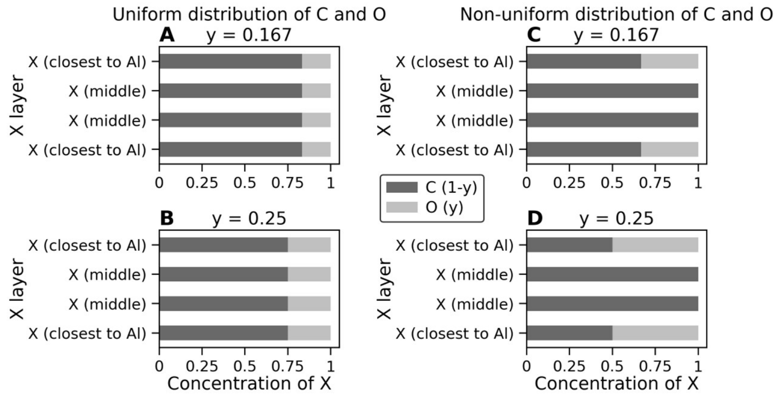

Figure S6. Schematic illustration of the considered distributions of C and O. (**A, B**) Uniform and (**C, D**) non-uniform, i.e., site dependent, distribution of C and O for different concentrations y of oxygen O in $M_5Al(C_{1-y}O_{y})_4$ and $(M'_{1-x}M''_{x})_5Al(C_{1-y}O_{y})_4$.

Stability

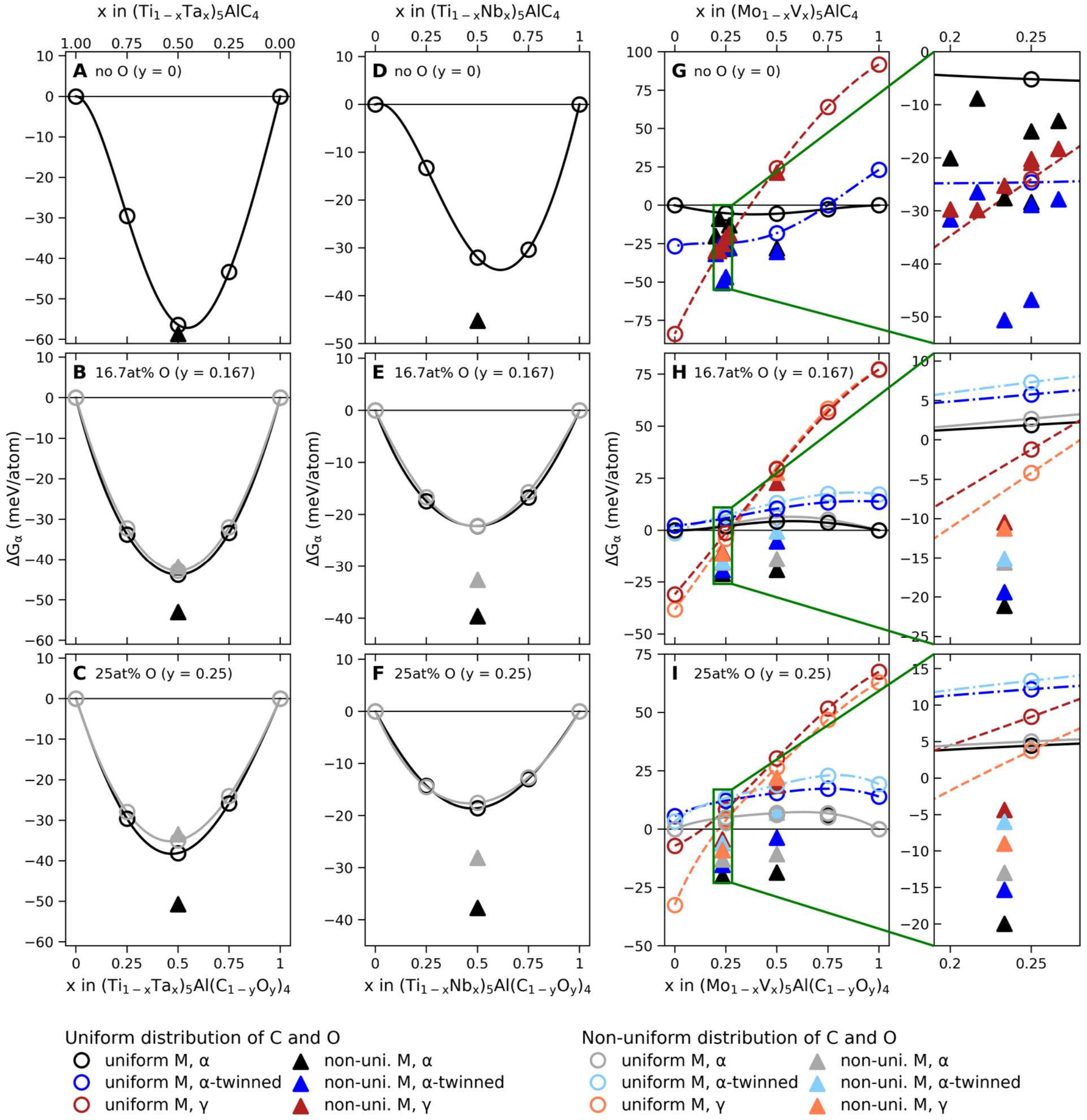

Figure S7. Relative stability in terms of isostructural formation enthalpy, i.e., with respect to total energy of α-stacking of M_5AlC_4 or $M_5Al(C_{1-y}O_y)_4$, evaluated at 0 K for $(Ti_{1-x}Ta_x)_5Al(C_{1-y}O_y)_4$ with (**A**) no oxygen, (**B**) 16.7 at.% oxygen and (**C**) 25 at.% oxygen, $(Ti_{1-x}Nb_x)_5Al(C_{1-y}O_y)_4$ with (**D**) no oxygen, (**E**) 16.7 at.% oxygen and (**F**) 25 at.% oxygen, and $(Mo_{1-x}V_x)_5Al(C_{1-y}O_y)_4$ with (**G**) no oxygen, (**H**) 16.7 at.% oxygen and (**I**) 25 at.% oxygen. Inset of (G, H, I) shows data around $(Mo_{0.75}V_{0.25})_5AlC_4$ for multiple structures with different distributions of Mo and V.

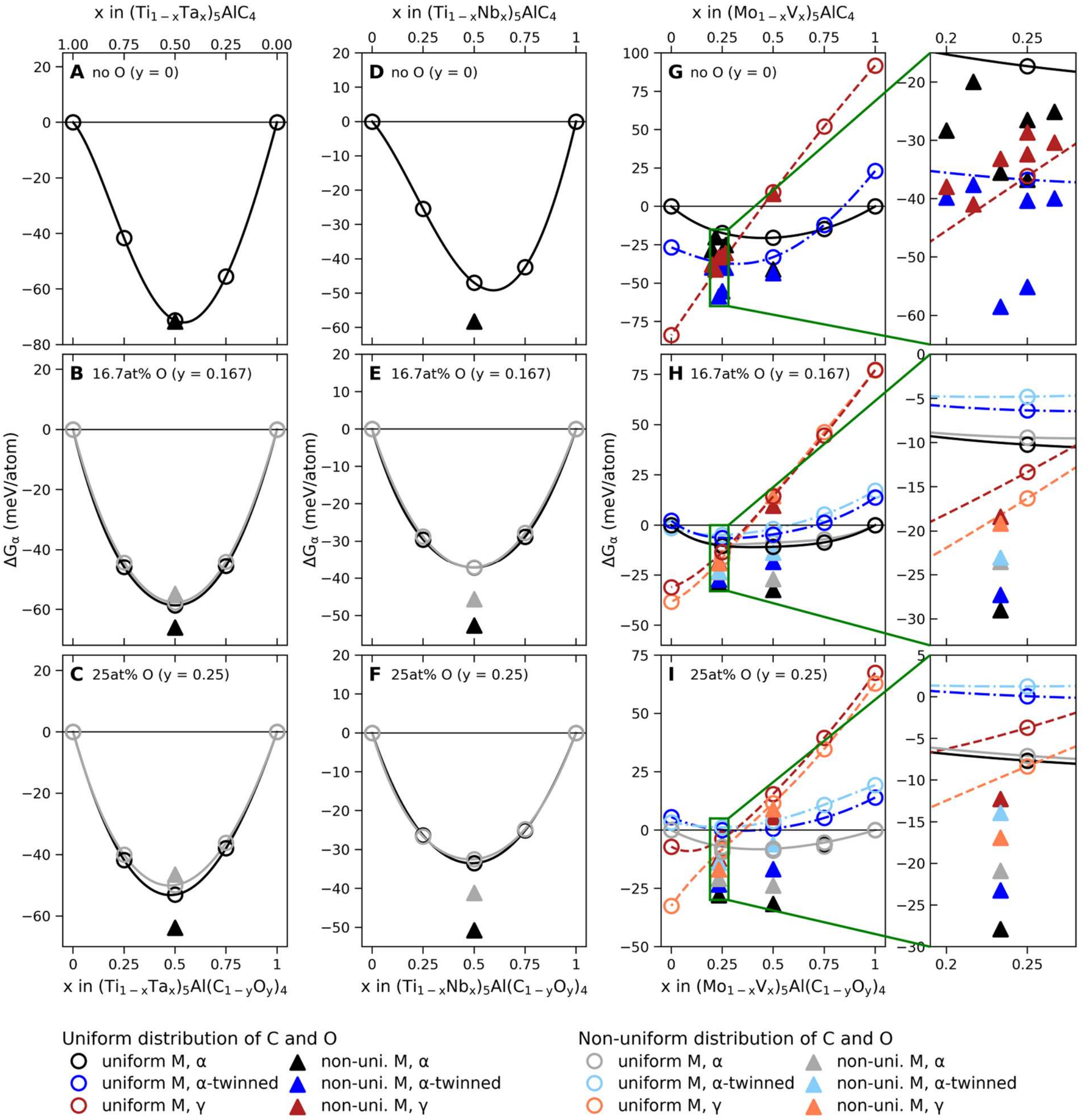

Figure S8. Relative stability in terms of isostructural formation free energy, i.e., with respect to total energy of α-stacking of M_5AlC_4 or $M_5Al(C_{1-y}O_y)_4$, evaluated at 500 K for $(Ti_{1-x}Ta_x)_5Al(C_{1-y}O_y)_4$ with (**A**) no oxygen, (**B**) 16.7 at.% oxygen and (**C**) 25 at.% oxygen, $(Ti_{1-x}Nb_x)_5Al(C_{1-y}O_y)_4$ with (**D**) no oxygen, (**E**) 16.7 at.% oxygen and (**F**) 25 at.% oxygen, and $(Mo_{1-x}V_x)_5Al(C_{1-y}O_y)_4$ with (**G**) no oxygen, (**H**) 16.7 at.% oxygen and (**I**) 25 at.% oxygen. Inset of (G, H, I) shows data around $(Mo_{0.75}V_{0.25})_5AlC_4$ for multiple structures with different distributions of Mo and V.

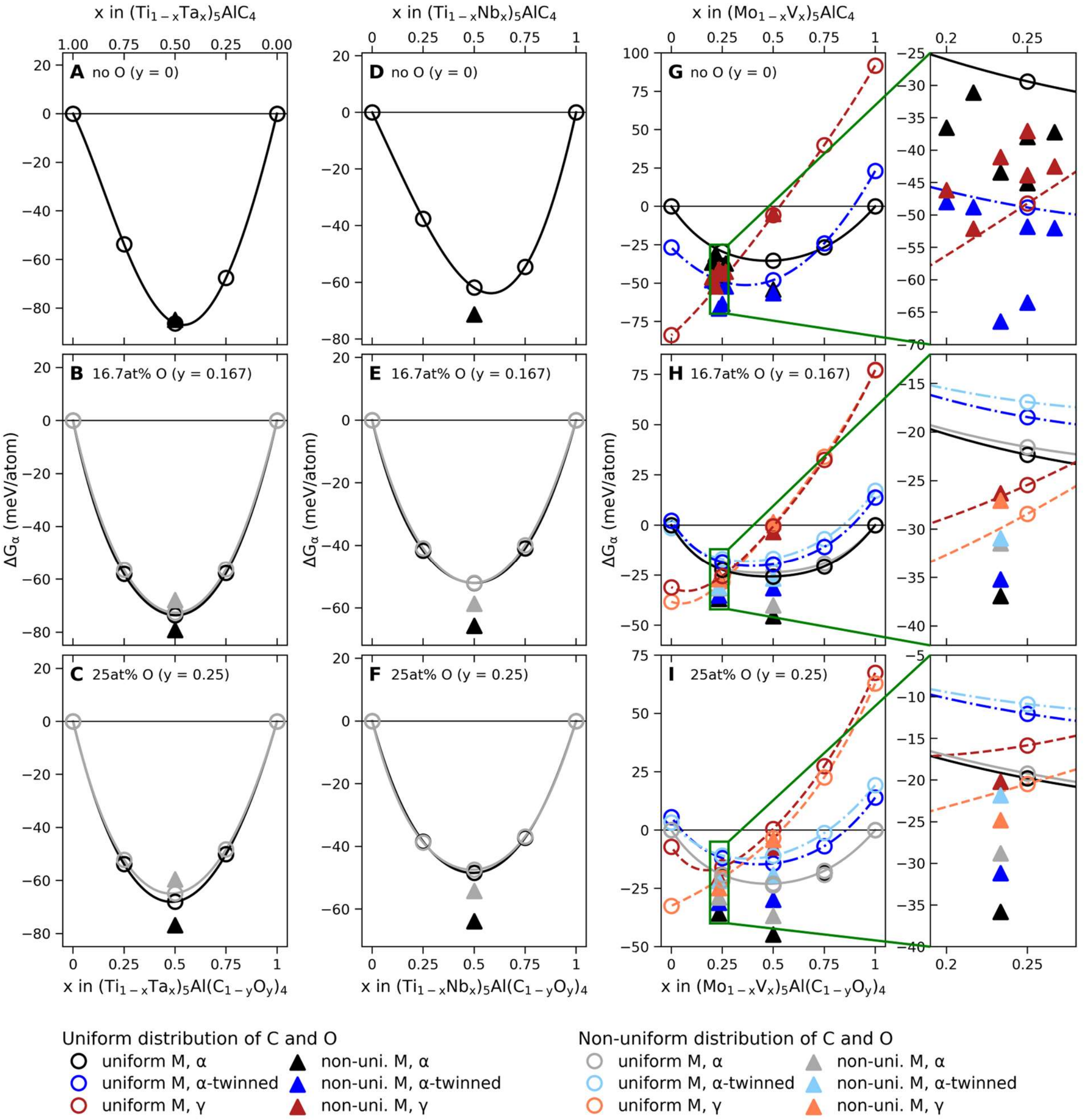

Figure S9. Relative stability in terms of isostructural formation free energy, i.e., with respect to total energy of α-stacking of M_5AlC_4 or $M_5Al(C_{1-y}O_y)_4$, evaluated at 1000 K for $(Ti_{1-x}Ta_x)_5Al(C_{1-y}O_y)_4$ with (**A**) no oxygen, (**B**) 16.7 at.% oxygen and (**C**) 25 at.% oxygen, $(Ti_{1-x}Nb_x)_5Al(C_{1-y}O_y)_4$ with (**D**) no oxygen, (**E**) 16.7 at.% oxygen and (**F**) 25 at.% oxygen, and $(Mo_{1-x}V_x)_5Al(C_{1-y}O_y)_4$ with (**G**) no oxygen, (**H**) 16.7 at.% oxygen and (**I**) 25 at.% oxygen. Inset of (G, H, I) shows data around $(Mo_{0.75}V_{0.25})_5AlC_4$ for multiple structures with different distributions of Mo and V.

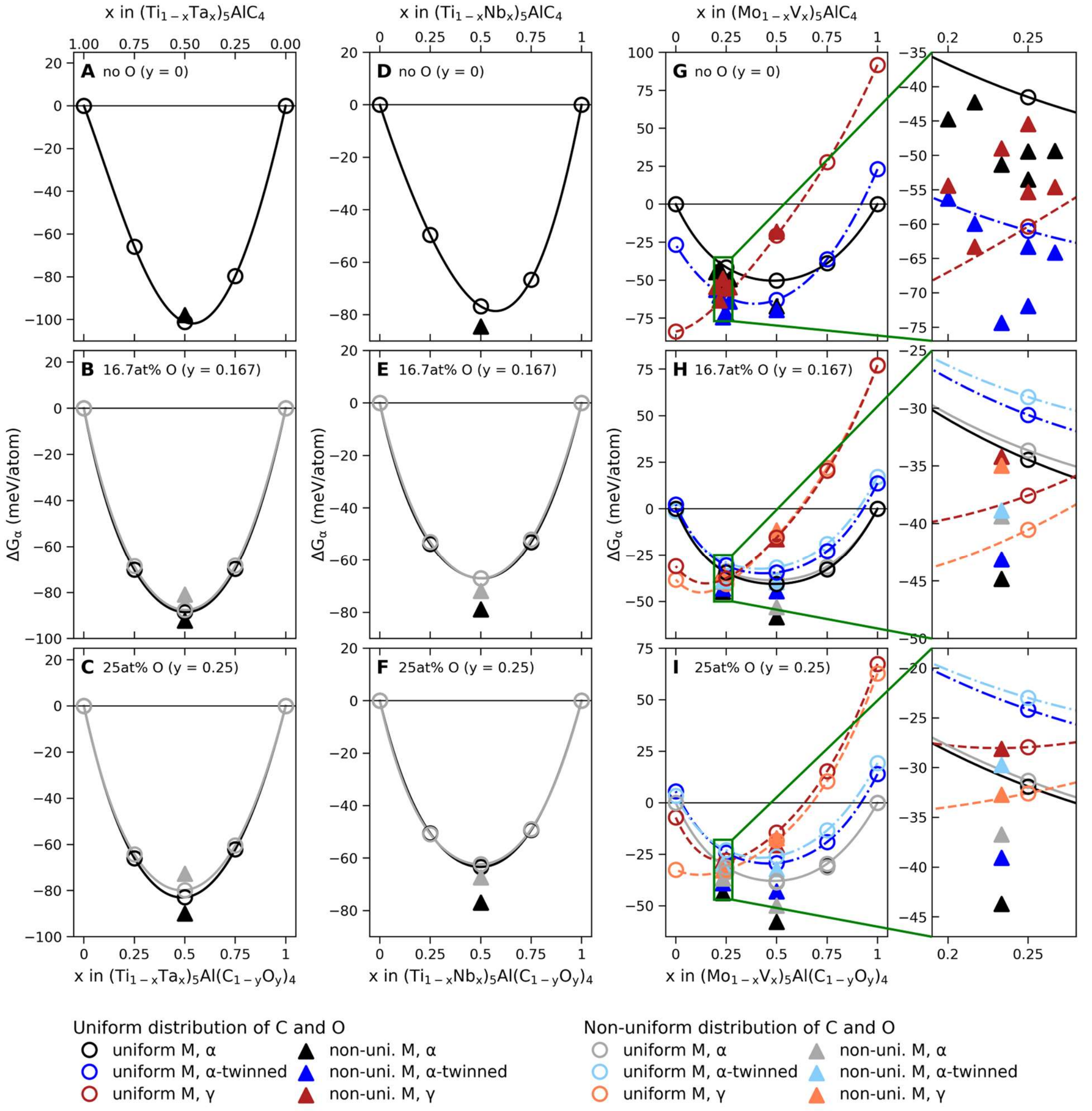

Figure S10. Relative stability in terms of isostructural formation free energy, i.e., with respect to total energy of α-stacking of M_5AlC_4 or $M_5Al(C_{1-y}O_y)_4$, evaluated at 1500 K for $(Ti_{1-x}Ta_x)_5Al(C_{1-y}O_y)_4$ with (**A**) no oxygen, (**B**) 16.7 at.% oxygen and (**C**) 25 at.% oxygen, $(Ti_{1-x}Nb_x)_5Al(C_{1-y}O_y)_4$ with (**D**) no oxygen, (**E**) 16.7 at.% oxygen and (**F**) 25 at.% oxygen, and $(Mo_{1-x}V_x)_5Al(C_{1-y}O_y)_4$ with (**G**) no oxygen, (**H**) 16.7 at.% oxygen and (**I**) 25 at.% oxygen. Inset of (G,H,I) shows data around $(Mo_{0.75}V_{0.25})_5AlC_4$ for multiple structures with different distributions of Mo and V.

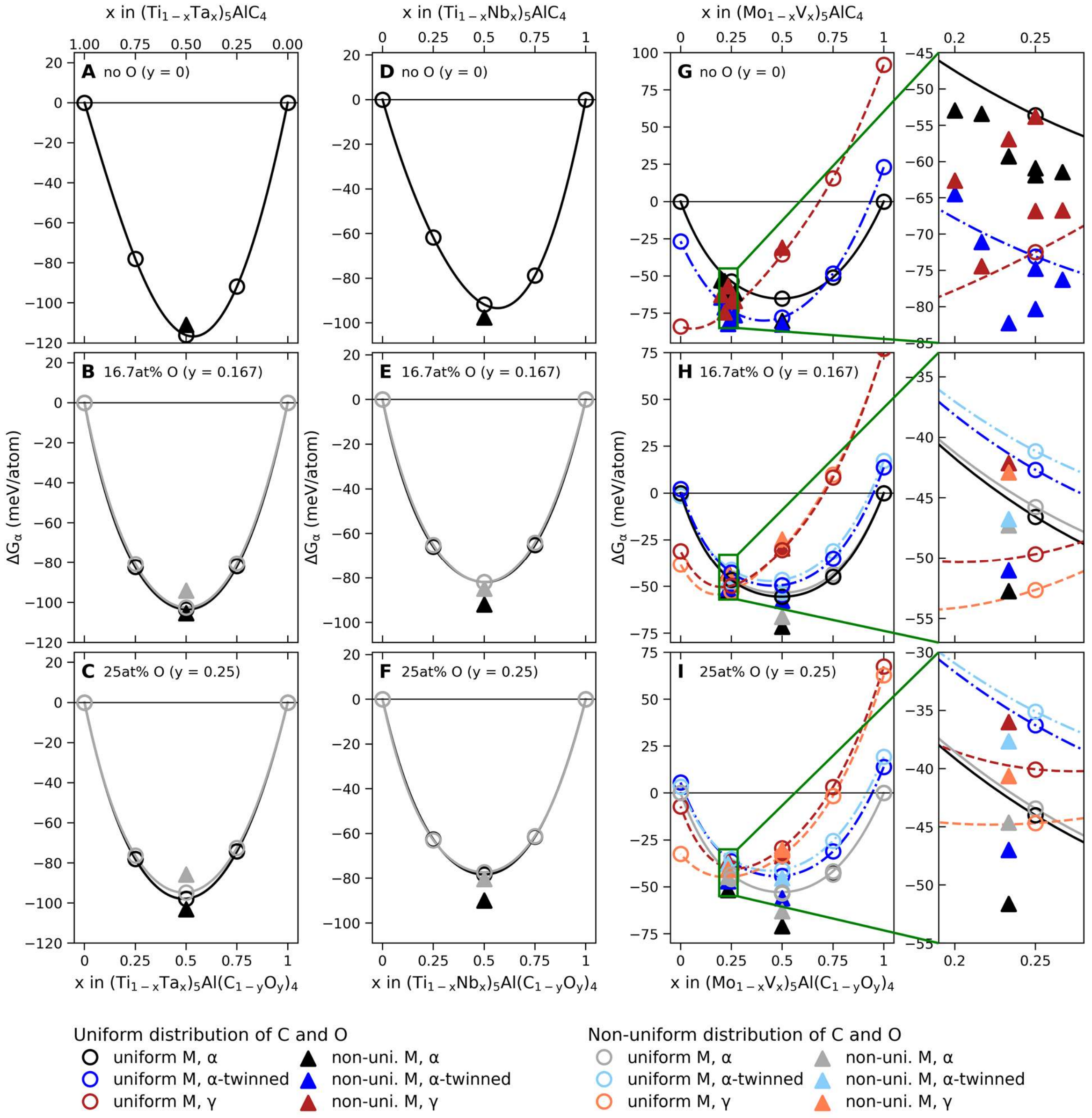

Figure S11. Relative stability in terms of isostructural formation free energy, i.e., with respect to total energy of α-stacking of M_5AlC_4 or $M_5Al(C_{1-y}O_y)_4$, evaluated at 2000 K for $(Ti_{1-x}Ta_x)_5Al(C_{1-y}O_y)_4$ with (**A**) no oxygen, (**B**) 16.7 at.% oxygen and (**C**) 25 at.% oxygen, $(Ti_{1-x}Nb_x)_5Al(C_{1-y}O_y)_4$ with (**D**) no oxygen, (**E**) 16.7 at.% oxygen and (**F**) 25 at.% oxygen, and $(Mo_{1-x}V_x)_5Al(C_{1-y}O_y)_4$ with (**G**) no oxygen, (**H**) 16.7 at.% oxygen and (**I**) 25 at.% oxygen. Inset of (G,H,I) shows data around $(Mo_{0.75}V_{0.25})_5AlC_4$ for multiple structures with different distributions of Mo and V.

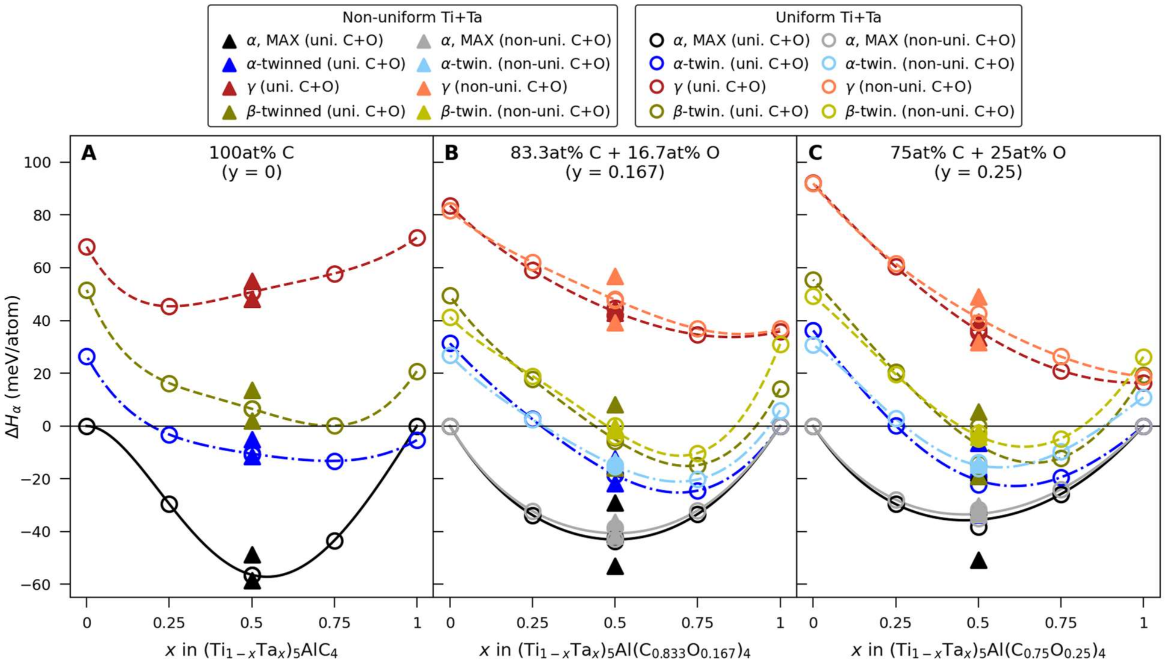

Figure S12. Relative stability in terms of isostructural formation enthalpy, i.e., with respect to total energy of α-stacking of M_5AlC_4 or $M_5Al(C_{1-y}O_y)_4$, evaluated at 0 K for $(Ti_{1-x}Ta_x)_5Al(C_{1-y}O_y)_4$.

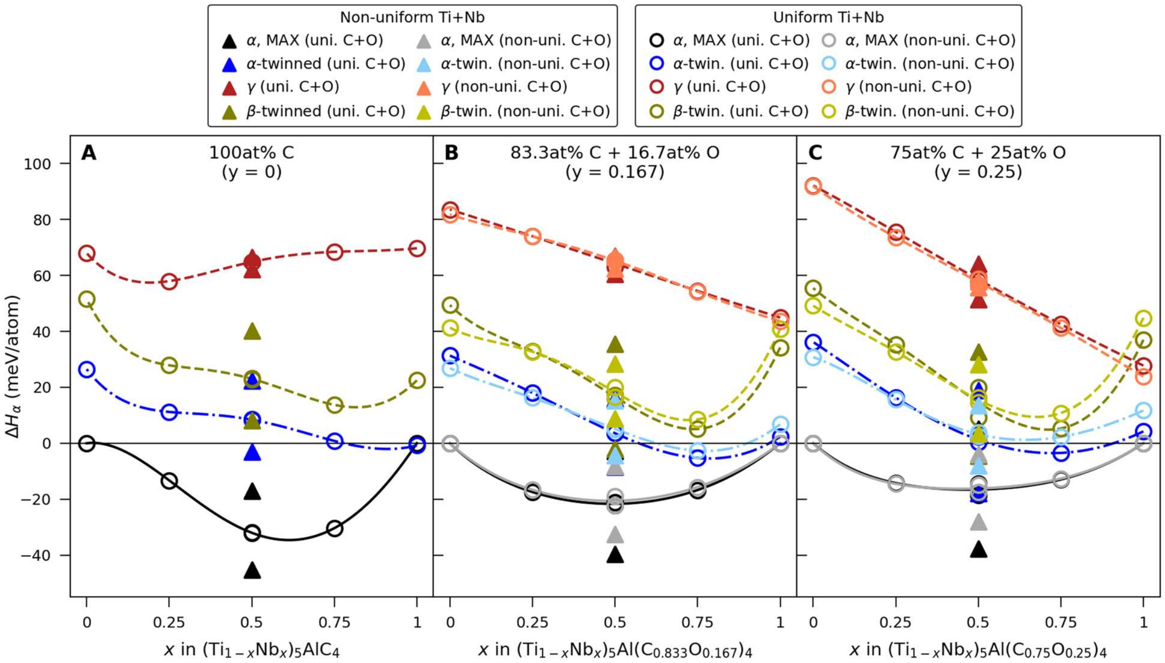

Figure S13. Relative stability in terms of isostructural formation enthalpy, i.e., with respect to total energy of α-stacking of M_5AlC_4 or $M_5Al(C_{1-y}O_y)_4$, evaluated at 0 K for $(Ti_{1-x}Nb_x)_5Al(C_{1-y}O_y)_4$.

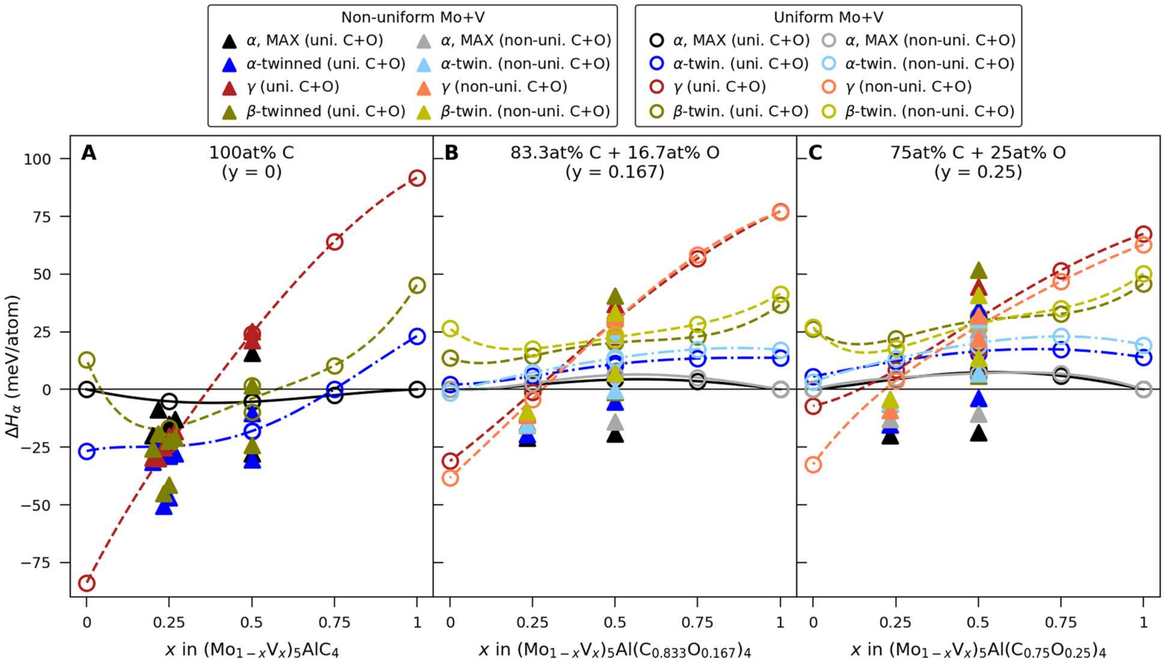

Figure S14. Relative stability in terms of isostructural formation enthalpy, i.e., with respect to total energy of α-stacking of M_5AlC_4 or $M_5Al(C_{1-y}O_y)_4$, evaluated at 0 K for $(Ti_{1-x}Ta_x)_5Al(C_{1-y}O_y)_4$.

Density of states

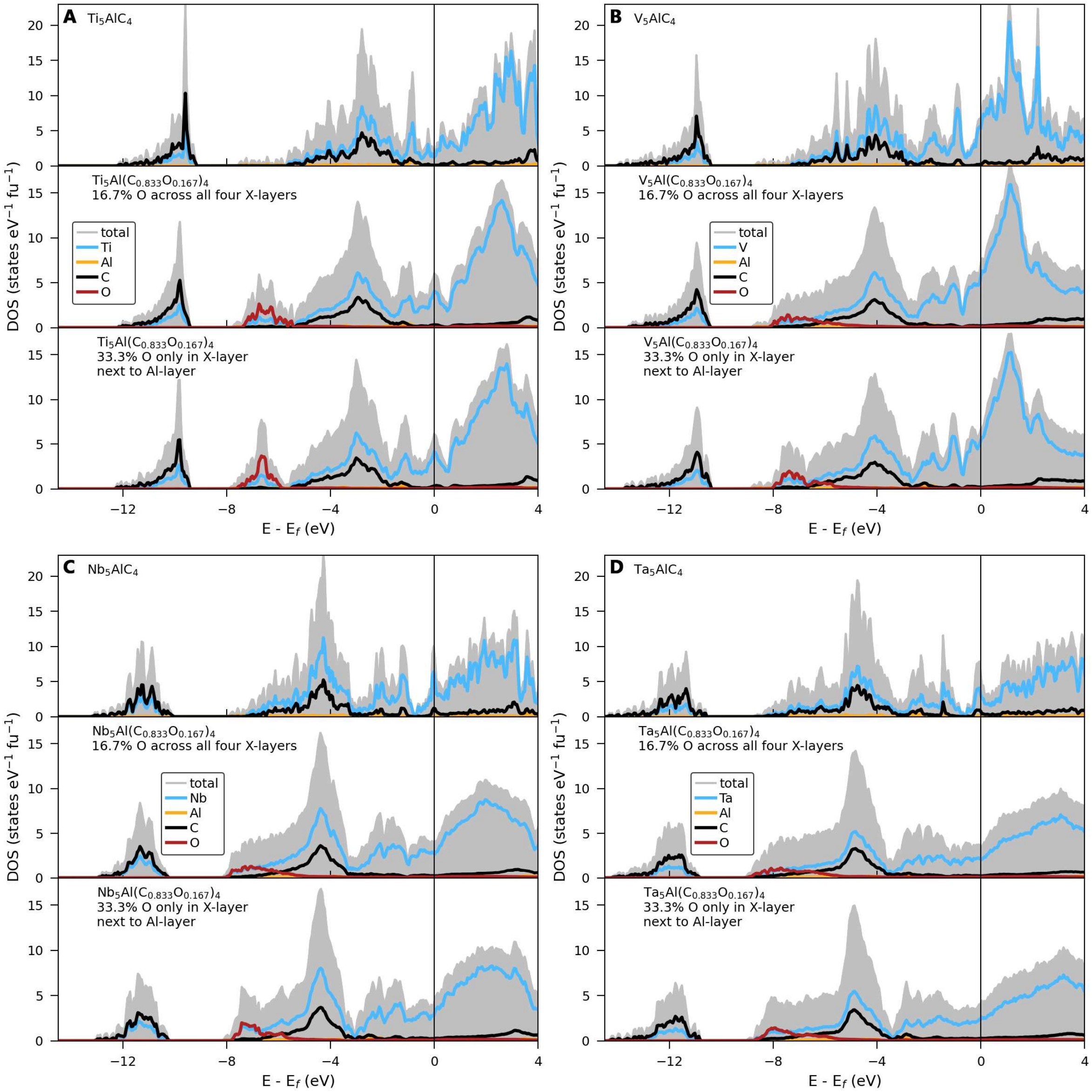

Figure S15. Calculated density of states (DOS) for $M_5Al(C_{1-y}O_y)_4$ with (**A**) M = Ti, (**B**) M = V, (**C**) M = Nb, and (**D**) M = Ta. Top panels with $y = 0$, i.e., no oxygen, mid-panels with $y = 0.167$ with oxygen uniformly distributed across all X-layers, and bottom panels for $y = 0.167$ with non-uniform distribution of oxygen, i.e., 33.3 % oxygen only in the outer X-layer closest to the Al layer.

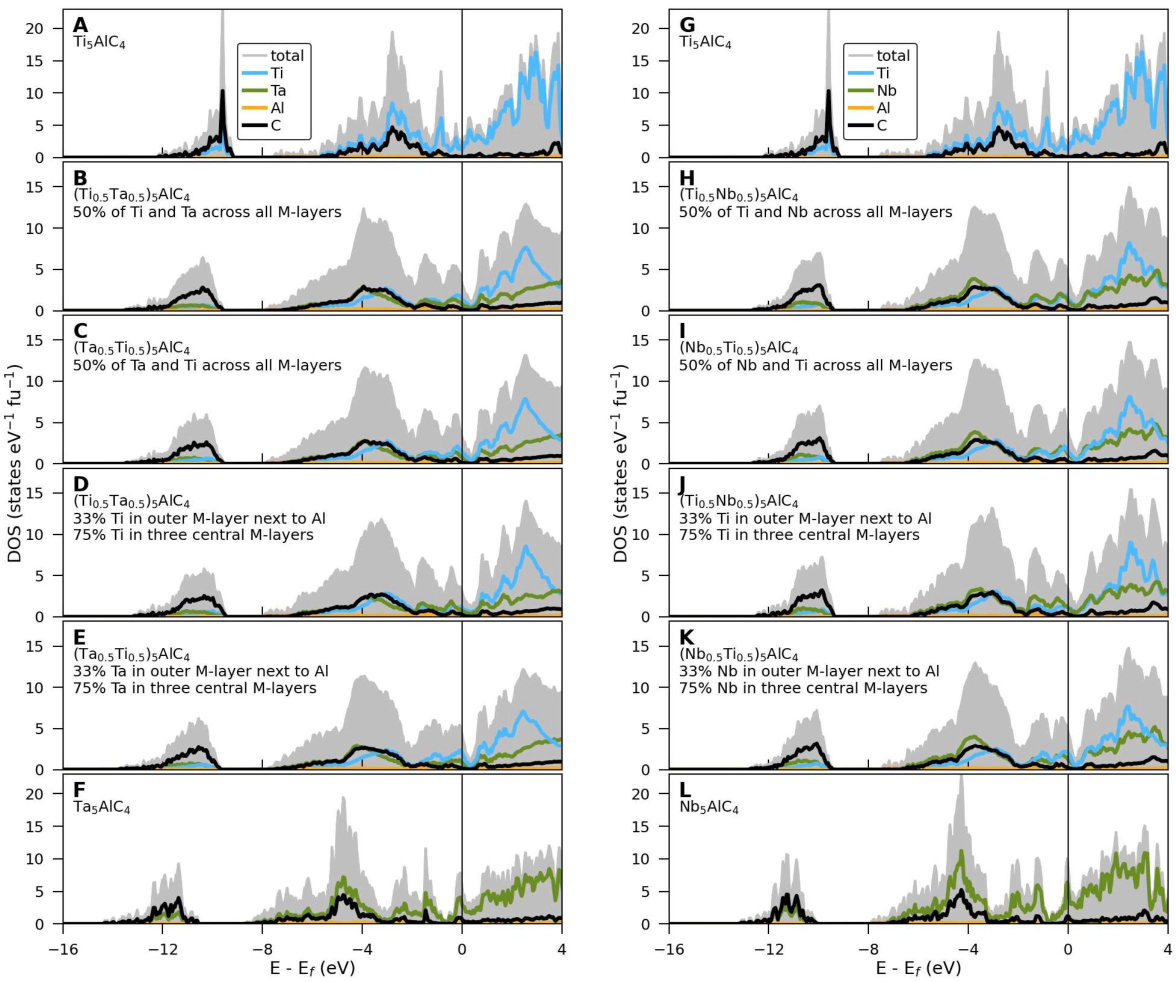

Figure S16. Calculated density of states (DOS) for (**A** to **F**) $(Ti_{1-x}Ta_x)_5AlC_4$ and (**G** to **L**) $(Ti_{1-x}Nb_x)_5AlC_4$ with uniform and non-uniform distribution of metals.

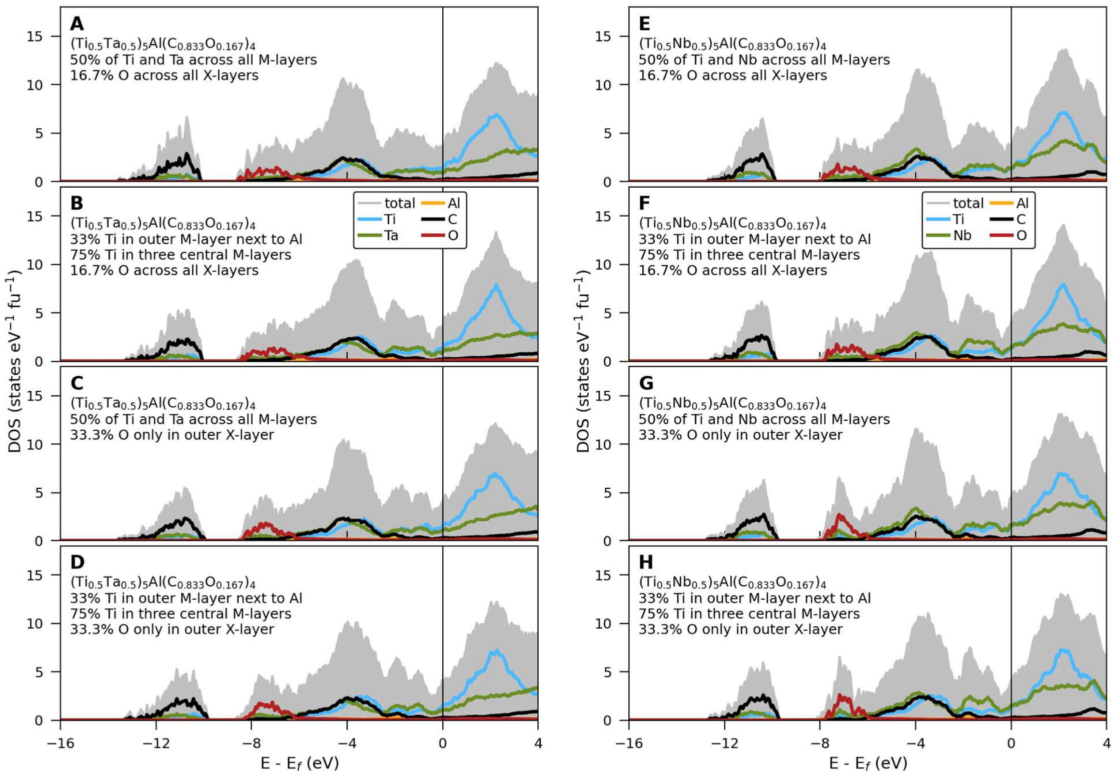

Figure S17. Calculated density of states (DOS) for (**A** to **D**) $(Ti_{1-x}Ta_x)_5Al(C_{1-y}O_y)_4$ and (**E** to **H**) $(Ti_{1-x}Nb_x)_5Al(C_{1-y}O_y)_4$ for $y = 0.167$ with uniform and non-uniform distribution of oxygen and metals.

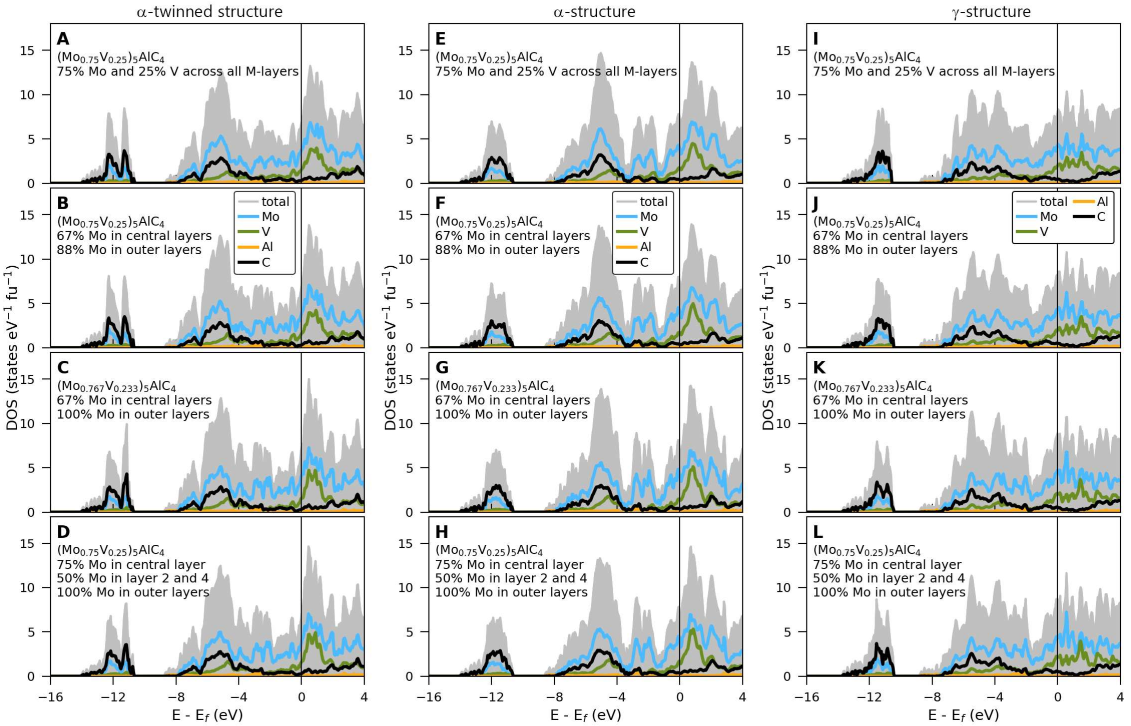

Figure S18. Calculated density of states (DOS) for $(Mo_{1-x}V_x)_5AlC_4$ in (**A** to **D**) α-twinned structure, (**E** to **H**) α structure, and (**I** to **L**) γ structure. Panels A, E, and I represent uniform distribution of Mo and V across all M-layers corresponding to **Figure S5A** and the three bottom rows different non-uniform distributions of Mo and V with (B, F, J) corresponding to **Figure S5G,** (C, G, K) corresponding to **Figure S5J**, and (D, H, L) corresponding to **Figure S5K**.

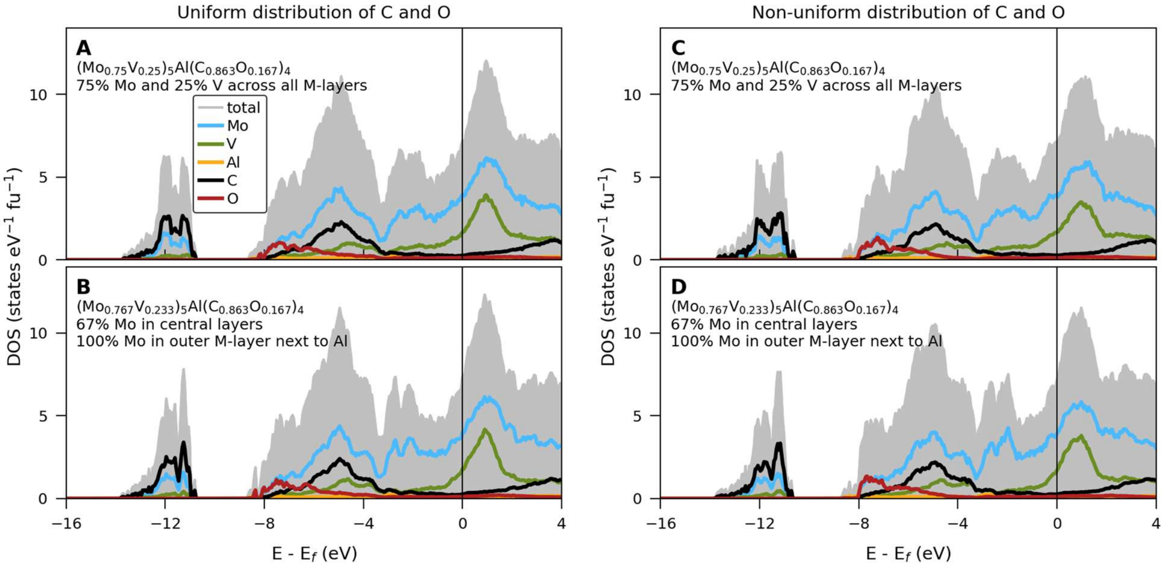

Figure S19. Calculated density of states (DOS) for α-twinned structure of $(Mo_{1-x}V_x)_5Al(C_{0.833}O_{0.167})_4$ with (**A**, **B**) uniform distribution of C and O across all X-layers corresponding to **Figure S6A** and (**C**, **D**) non-uniform distribution of C and O corresponding to **Figure S6C**. Panels (A, C) represent uniform distribution of Mo and V across all M-layers corresponding to **Figure S6A** and (B, D) non-uniform distribution of Mo and V corresponding to **Figure S6J**.
